# Genomic context-dependent histone H3K36 methylation by three *Drosophila* methyltransferases and implications for dedicated chromatin readers

**DOI:** 10.1093/nar/gkaf202

**Published:** 2025-04-01

**Authors:** Muhunden Jayakrishnan, Magdalena Havlová, Václav Veverka, Catherine Regnard, Peter B Becker

**Affiliations:** Molecular Biology Division, Biomedical Center, Ludwig-Maximilians-Universität, 82152 Munich, Germany; Institute of Organic Chemistry and Biochemistry (IOCB) of the Czech Academy of Sciences, 166 10 Prague, Czech Republic; Institute of Organic Chemistry and Biochemistry (IOCB) of the Czech Academy of Sciences, 166 10 Prague, Czech Republic; Department of Cell Biology, Faculty of Science, Charles University, 128 44 Prague, Czech Republic; Molecular Biology Division, Biomedical Center, Ludwig-Maximilians-Universität, 82152 Munich, Germany; Molecular Biology Division, Biomedical Center, Ludwig-Maximilians-Universität, 82152 Munich, Germany

## Abstract

Methylation of histone H3 at lysine 36 (H3K36me3) marks active chromatin. The mark is interpreted by epigenetic readers that assist transcription and safeguard chromatin fiber integrity. In *Drosophila*, the chromodomain protein MSL3 binds H3K36me3 at X-chromosomal genes to implement dosage compensation. The PWWP-domain protein JASPer recruits the JIL1 kinase to active chromatin on all chromosomes. Because depletion of K36me3 had variable, locus-specific effects on the interactions of those readers, we systematically studied K36 methylation in a defined cellular model. Contrasting prevailing models, we found that K36me1, K36me2, and K36me3 each contribute to distinct chromatin states. Monitoring the changing K36 methylation landscape upon depletion of the three methyltransferases Set2, NSD, and Ash1 revealed local, context-specific methylation signatures. Each methyltransferase governs K36 methylation in dedicated genomic regions, with minor overlaps. Set2 catalyzes K36me3 predominantly at transcriptionally active euchromatin. NSD places K36me2/3 at defined loci within pericentric heterochromatin and on weakly transcribed euchromatic genes. Ash1 deposits K36me1 at putative enhancers. The mapping of MSL3 and JASPer suggested that they bind K36me2 in addition to K36me3, which was confirmed by direct affinity measurement. This dual specificity attracts the readers to a broader range of chromosomal locations and increases the robustness of their actions.

## Graphical Abstract

**Graphical Abstract:**
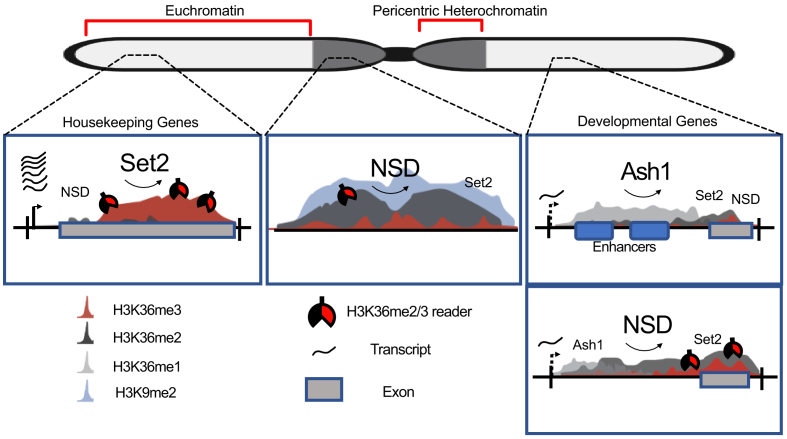

## Introduction

Histone modifications highlight functional chromatin states. Particular chemical modifications inform about whether large chromatin domains are permissive or repressive to transcription and demarcate regulatory sequences within these domains. Combinations of modifications, most prominently acetylation and methylation of specific histone lysines, constitute epigenomic signatures that allow interpreting eukaryotic genomes [1–3].

A prominent example of a histone modification that is associated with a specific function is the trimethylation of lysine 36 of histone H3 (H3K36me3; K36me3 for short), which maps to bodies of transcribed genes. This mark correlates to active transcription in all eukaryotes from yeast to mammals. This methylation signals the “transcribed” state of chromatin to localize a wide variety of processes that go along with active transcription, such as splicing [4, 5], nucleosome turnover [6], deacetylation [7, 8], DNA repair [9–11], RNA methylation [12], establishment of DNA methylation [13, 14], and counteraction of facultative heterochromatin spreading [15, 16], as well as regulation of *S*-adenosyl methionine flux [17, 18]. For example, K36me3 recruits enzymes to reinstate the integrity of the nucleosome fiber during transcription and to repress cryptic, intragenic promoters in yeast [19]. In human cells, H3K36me3 can recruit DNA methyltransferases and decreased H3K36me3 is associated with increased cryptic transcription during aging [20] as well as splicing defects [21]. K36me3 serves as an epigenetic mark that targets dosage compensation to X-linked genes in *Drosophila* males [22].

*Drosophila* has only two H3 variants and replacement of replication-dependent H3.2 or replication-independent H3.3 with non-methylatable K36R mutants has allowed to study the role of K36 in development [23–25]. H3.2K36 accounts for the majority of H3K36me3 and is essential for viability, and its loss mostly affects post-transcriptional processing of messenger RNA (mRNA) [26]. Intriguingly, the same study showed that splicing and transcription are only mildly affected, with observed defects frequently not restricted to K36me3 regions [26], contrasting previous reports regarding involvement of K36me3 in splicing [21] and repression of spurious transcription [19]. In contrast, H3.3K36 accounts for ∼20% of H3K36me3 and supports longevity [27]. The two variants may be redundant under certain contexts, e.g. during X-chromosome dosage compensation [28].

The effects of histone methylation are usually mediated by “reader” proteins, i.e. proteins that contain domains that recognize and bind specifically to methylated lysines. As subunits of larger enzyme complexes, these reader proteins tether epigenetic regulators to specific chromatin regions [29]. Reader domains for H3K36 methylation include PWWP [30, 31], chromo [32, 33], and tudor domains [34]. We are interested in two *Drosophila* proteins for which K36me3 seems to be the main determinant for genomic distribution: MSL3, the reader subunit of the male-specific lethal dosage compensation dosage compensation complex (DCC) that boosts the transcription on the X chromosome in male flies [35], and JASPer, the targeting subunit of the JJ complex responsible for interphase H3S10 phosphorylation, which is also enriched on the male X chromosome [36].

Lysines may be modified by one, two, or three methyl groups. Whereas K36me3 has been extensively studied, K36me1/2 is less understood. The prevailing view, mainly based on characterization of the distribution of the H3K36 methylation marks in yeast, was that K36me3 corresponded to the true functional state, while K36me1/2 were mostly methylation intermediates.

Histone methylation profiles are established by dedicated histone methyltransferases (HMTs). In mammals, H3K36 HMTs are to some extent redundant. SetD2 catalyzes K36me1/2/3, whereas NSD1, NSD2, and ASH1L catalyze K36me2 and NSD3 catalyzes K36me1 [17]. In *Drosophila*, only three evolutionary conserved enzymes methylate K36, namely Set2 (alias dHypb), NSD (alias dMes-4), and Ash1. Set2 (orthologous to mammalian SetD2) is an interactor of the elongating RNA polymerase II and is thought to catalyze all K36me3 [35, 37]. NSD (orthologous to mammalian NSD1/2/3) has been reported to localize to both eu- and heterochromatin and is known predominantly as a mono- and dimethyltransferase [37, 38]. Finally, Ash1 (orthologous to mammalian Ash1L) is described as dimethyltransferase whose role is developmentally restricted to a few hundred genomic loci to counteract Polycomb repression [39–41]. Interestingly, some of the effects observed upon deletion of Set2 or other H3K36 methyltransferases have not been recapitulated by histone replacement by their non-methylatable counterparts. For example, while Set2 deletion leads to imbalanced gene expression of X-linked genes in male flies, the effect is absent in flies with H3.2K36R replacement [28]. A similar study also showed that excessive Polycomb repression of *HOX* genes observed upon Ash1 loss is not recapitulated in K36R flies [25]. These observations may hint at the possibility that some of the effects attributed to H3K36 methylation may be due to methylation of other non-histone proteins.

According to the prevailing model of the division of labor, NSD first “premodifies” chromatin with K36me1/2, which then serves as a substrate for Set2 to cotranscriptionally generate K36me3, establishing a linear relationship between the two enzymes [37, 42, 43]. Ash1 is believed to function independently of the Set2–NSD tandem [41, 44]. This simple view is currently being challenged by several independent observations. In *Drosophila*, K36me3, K36me2, and K36me1 are differently distributed, implying distinct functions for each methylation state [2, 28]. Furthermore, the transcriptional and developmental consequences of NSD deletion are distinct from those of Set2 deficiency [25, 28]. For instance, Set2-null flies die during late larval stage, while NSD-null flies are viable and fertile (albeit with minor defects), arguing against an exclusive linear relationship of the two enzymes toward generating the trimethylated state [25, 35, 37]. Interpretation of these organismal phenotypes is complicated by suspected cell type-specific and developmental effects.

To more systematically evaluate the function of the three *Drosophila* HMTs and to characterize the different patterns of the K36 methylation states at high resolution, we resorted to a defined tissue culture model. We mapped the individual K36 methylation states at high resolution using an enhanced chromatin immunoprecipitation (ChIP) protocol that combines the resolution of micrococcal nuclease (MNase) digestion with solubilization of heterochromatin by mild shearing [36]. We describe the consequences of systematic HMT depletion, individual and in combination, on the K36 methylation landscape and highlight differences in local HMT dependences. In contrast to the prevailing model, we find that the three enzymes establish distinct methylation states at defined genomic regions.

We also monitored the distribution of the two K36me3 reader proteins MSL3 and JASPer upon HMT depletion and showed that reduction of K36me3 did not lead to a proportional reduction in reader’s binding. We propose that the robustness of the reader’s recruitment is at least in part due to their ability to bind both K36me2 and K36me3 combined with threshold effects and influence of the chromatin context.

Our systematic study uncovers an unexpected complexity of the three different H3K36 methylation states, which likely have different functions depending on the local chromatin context.

## Materials and methods

### Cell culture and RNAi

S2-DRSC (DGRC stock #), Kc167 (DGRC stock #) cells were cultured in Schneider’s *Drosophila* Medium (Thermo Fisher), supplemented with 10% heat-inactivated fetal bovine serum (FBS; Sigma–Aldrich), 100 units/ml penicillin, and 0.1 mg/ml streptomycin (Sigma–Aldrich) at 26°C. RNA interference (RNAi) against target genes was performed in either large scale (for ChIP-seq) or small scale (western blots, immunofluorescence staining, RNA extraction).

*Large scale*: 1 × 10^6^ S2 cells were seeded per well of a six-well dish (Cellstar) (two wells per RNAi condition). Cells were washed once in serum-free medium followed by treatment with 10 μg double-stranded RNA (dsRNA)/well/RNAi target in 1 ml serum-free medium. Two different dsRNAs were used for each target to improve RNAi efficiency. Cells were incubated for 10 min at room temperature (RT) with slight agitation and further 50 min at 26°C. Two volumes of complete growth medium were added and cells were incubated for 5 days at 26°C. At day 5, cells were resuspended and counted. 2 × 10^7^ cells per RNAi condition were transferred to a 75-cm^2^ flask for a second round of dsRNA treatment (80 μg dsRNA/flask/RNAi target) in 8 ml serum-free medium and incubated as mentioned earlier. At day 10, cells were counted and processed for ChIP-seq. For Ash1, to improve knockdown efficiency, three rounds of knockdowns were performed for 12 days as described by Schwartz *et al.* [45].

*Small scale*: 3 × 10^5^ S2 cells were seeded per well of a 12-well dish (Cellstar). Once adhered, cells were washed once in serum-free medium followed by treatment with 3–4 μg dsRNA/well/RNAi target in 300 μl serum-free medium. Cells were incubated for 10 min at RT with slight agitation and further 50 min at 26°C. Two volumes of complete growth medium were added and cells were incubated for 5 days at 26°C. At day 5, cells were resuspended and counted. 1.5 × 10^6^ cells per RNAi condition were transferred to a six-well dish for a second round of dsRNA treatment (10 μg dsRNA/flask/RNAi target) in 1 ml serum-free medium and incubated as mentioned earlier. At day 10, cells were counted and processed for western blot or immunofluorescence.

dsRNA was generated by *in vitro* transcription (NEB T7 E2050s transcription kit) from polymerase chain reaction (PCR) products generated by the following forward and reverse primers (separated by comma):

- *gst* RNAi: TTAATACGACTCACTATAGGGAGAATGTCCCCTATACTAGGTTA,

TTAATACGACTCACTATAGGGAGAACGCATCCAGGCACATTG.

The sequence of *Schistosoma japonicum* GST was amplified from pGEX-4T-1 (GE Healthcare).

- *gfp* RNAi: TTAATACGACTCACTATAGGGTGCTCAGGTAGTGGTTGTCG,

TTAATACGACTCACTATAGGGCCTGAAGTTCATCTGCACCA;

- Set2_#1 RNAi: TTAATACGACTCACTATA*G*GGAGAAAATCCTTGATTCCAAGCAA,

TTAATACGACTCACTATAGGGAGAAGTGGTTTCTACATTTTCGT;

- Set2_#2 RNAi: TTAATACGACTCACTATAGGGAGACACGGCTTGAGATTGCTACA,

TTAATACGACTCACTATAGGGAGACATGGACATGCTTTTGTTGG;

- NSD_#1 RNAi: TTAATACGACTCACTATAGGGAGACGCGAATTCCTGAGCACGGACGCGCACTC,

TTAATACGACTCACTATAGGGAGACGCTCTAGATGGACACACGCTGTTGTTGCTGTTT;

- NSD_#2 RNAi: TTAATACGACTCACTATAGGGAGACCCTCCTCTGTGAGCATCGA,

TTAATACGACTCACTATAGGGAGAACAACGTTTTCGTACGTCTGG;

- Ash1_#1 RNAi: TTAATACGACTCACTATAGGGAGACTTTGTGGCCAGGACCAATCAA,

TTAATACGACTCACTATAGGGAGACAGGCAAGGGATCGTGCTCGGT;

- Ash1_#2 RNAi: CTAATACGACTCACTATAGGGAGGCAGTGCCATGGAGACCC,

CTAATACGACTCACTATAGGGAGCAACACCCAGCAGCGTCC.

*Drosophila virilis* 79f7Dv3 cells [36] were cultured in Schneider’s *Drosophila* Medium (Thermo Fisher), supplemented with 5% heat-inactivated FBS (Sigma–Aldrich), 100 units/ml penicillin, and 0.1 mg/ml streptomycin (Sigma–Aldrich) at 26°C.

### Immunofluorescence microscopy and analysis

Immunofluorescence microscopy (IFM) analysis of S2 cells was performed as described in [46]. Briefly, cells after RNAi were seeded on poly-l-lysine [Sigma #P8920, 0.01% (w/v) final concentration]-coated coverslips and allowed to adhere for 1 h. Coverslips were then fixed, permeabilized, blocked, and incubated in primary antibodies overnight at appropriate dilutions (see table in the “Antibodies” section). Secondary antibody staining was performed the next day after which coverslips were incubated in DAPI and sealed onto glass slides with Vectashield mounting reagent.

Confocal images were acquired on a Leica TCS SP8 with a 63×/1.4 NA oil-immersion objective. Image stacks were recorded at 100 Hz scan speed with a pixel size of 350 nm (or 50 nm for Supplementary Fig. S1B) and *z*-step size of 300 nm. Pinhole was set to 1 au (580 nm reference wavelength). Fluorescence signals were recorded sequentially to avoid channel crosstalk. Further image processing and maximum intensity projections were done in Fiji [47]. CellProfiler [48] was used to quantify histone modification immunostaining signals within DAPI defined regions (pipeline provided) and further plotted on R.

### Western blot

2–3 × 10^6^ RNAi-treated cells were pelleted and lysed in 1× Laemmli buffer at a concentration of 25 000 cells/μl. Samples were denatured at 95°C for 10 min. Six to eight microliters of lysate per sample was electrophoresed on SDS ServaGel TGPrimer (14% gel for histone modifications, 8% gel for other proteins) for 1.5–2 h at 180 V. Proteins were transferred to Amersham™ Protran™ 0.45-μm nitrocellulose blotting membrane for 1.5 h at 300–400 mA in either regular transfer buffer (20% MeOH, 25 mM Tris, 192 mM glycine) or high molecular weight transfer buffer for methyltransferases (10% MeOH, 0.037% SDS, 25 mM Tris, 192 mM glycine). Membranes were blocked with 3% bovine serum albumin (BSA) for 1 h at RT. The membrane was incubated with primary antibody (see table in the “Antibodies” section) overnight at 4°C in 3% BSA PBS washed thrice with PBS-T (1× phosphate-buffered saline, 0.1% Tween 20) and incubated with secondary antibody in PBS-T for 1 h at RT. Images were acquired and quantified using the LICOR Odyssey CLx. For all quantifications, we verified that the signal is linear in the range of loading used (not shown). However, due to blot-to-blot variability, the normalized signals should be interpreted as an estimate rather than an absolute quantification of protein levels.

### RNA isolation, cDNA synthesis, and RT-qPCR

Total RNA was extracted from 1.5 × 10^6^ cells using the RNeasy Mini Kit (Qiagen) according to the manufacturer’s instructions. Complementary DNA (cDNA) was synthesized from 500 ng of total RNA using Superscript III First Strand Synthesis System (Invitrogen, Cat. No. 18080-051, random hexamer priming) and following standard protocol. cDNA was diluted 1:100; quantitative PCR (qPCR) reaction was assembled using Fast SYBR Green Mastermix (Applied Biosystem, Cat. No. 4385612) and ran on a LightCycler 480 II (Roche) instrument. Primer efficiencies were calculated via serial dilutions. Primer sequences for Ash1 and 7sk (control) were obtained from [41] and [49], respectively.

### Chromatin immunoprecipitation after MNase treatment (MNase ChIP-seq) with spike-in

ChIP-seq on MNase-digested chromatin and sonicated chromatin was performed as previously described [36]. For spike-in ChIP-seq on MNase-digested chromatin in combination with mild sonication, S2 cells (∼1.2 × 10^8^ cells) after RNAi were harvested and cross-linked for 8 min by adding 1.1 ml of 10× fixing solution [50 mM HEPES, pH 8.0, 100 mM NaCl, 1 mM EDTA, 0.5 mM EGTA, and 10% (w/v) methanol-free formaldehyde] per 10 ml culture at RT. The reaction was stopped by adding glycine at 125 mM final concentration and incubating for 10 min on ice. Cells were washed twice in PBS before subsequent steps. For nuclei isolation, S2 cells were spiked with 5% (relative cell number) 79f7Dv3 fixed cells (processed as described for S2 cells) and resuspended in PBS supplemented with 0.5% (v/v) Triton X-100 and cOmplete EDTA-free Protease Inhibitor Cocktail (Roche) (PI), volume was adjusted to 7 × 10^7^ cells/ml, and cells incubated for 15 min at 4°C with end-over-end rotation. Nuclei were collected by centrifuging at 4°C for 10 min at 2000 × *g* and washed once in PBS. For chromatin fragmentation, nuclei were spun down at 4°C for 10 min at 2000 × *g*, resuspended in RIPA [10 mM Tris–HCl, pH 8.0, 140 mM NaCl, 1 mM EDTA, 1% (v/v) Triton X-100, 0.1% (v/v) SDS, 0.1% (v/v) sodium deoxycholate] supplemented with PI and 2 mM CaCl_2_ at 7 × 10^7^ cells/ml. These lysates were digested in 1 ml aliquots by adding 0.6 U MNase (Sigma–Aldrich), resuspended in EX-50 (50 mM KCl, 10 mM HEPES, pH 7.6, 1.5 mM MgCl_2_, 0.5 mM EGTA, 10% glycerol) at 0.6 U/μl, and incubated at 37°C for 35 min with slight agitation. The reaction was stopped by adding 10 mM EGTA and placing on ice. Digested chromatin was mildly sheared further with Covaris AFA S220 using 12 × 12 tubes at 50 W peak incident power, 20% duty factor, and 200 cycles per burst for 8 min at 5°C. We noticed that this step results in a more uniform solubilization of chromatin across the entire genome compared to protocols that use either MNase only [50] or shearing only [41, 51], based on comparison of respective chromatin input coverages, and thus is more suited for quantitative comparisons between eu- and heterochromatic ChIP signals. Subsequent steps were performed as described in [36]. Libraries were prepared with NEBNext Ultra II DNA Library Prep Kit for Illumina (NEB, E7645) and analyzed with either 2100 Bioanalyzer or TapeStation systems (Agilent). Libraries were sequenced on NextSeq1000 (Illumina) instrument yielding typically 20–25 million 150-bp paired-end reads per sample.

### CUT&RUN

CUT&RUN was performed as previously described [52] with the Rb α-H3K36me3 antibody diluted 1/500.

### Library preparation

For ChIP-seq samples, libraries were prepared using NEBNext Ultra II DNA Library with a starting ChIP DNA amount of 3–6 ng according to manufacturer’s instructions. All libraries were sequenced on an Illumina NextSeq1000 sequencer at the Laboratory of Functional Genomic Analysis (LAFUGA, Gene Center Munich, LMU). About 20 million paired-end reads were sequenced per sample for each of the ChIP samples.

### Antibodies

**Table:** 

Antibody	Western	IF	ChIP	Supplier/reference
Rb α-H3K36me1	1:5000	1:800	2 μl	Abcam, ab9048
Rb α-H3K36me2	1:5000	1:800	2 μl	Abcam, ab9049
Rb α-H3K36me3	1:5000	1:800	2 μl	Abcam, ab9050
Rb α-H4K16ac			2 μl	Millipore, 07-329
Ms α-H3K9me2			2 μl	Abcam, ab1220
Rb α-H3K27me3			2 μl	Millipore, 07-449
Rb α-H2AV	1:500			Börner and Becker [53]
Gp α-MSL3			3 μl	Albig *et al.* [36]
Gp α-JASPer			3 μl	Albig *et al.* [36]
Rb α-NSD	1:1000		5 μl	Bell *et al.* [37]
Ms α-NSD	1:50	1:10		Bell *et al.* [37]
Ms α-Set2	1:50			Bell *et al.* [37]
RBP1-S2ph (ePol)			2 μl	Abcam, ab5095
Lamin	1:1000			Gift from H. Saumweber

### Recombinant protein expression and purification

JASPer full-length sequence was cloned into pET-derived T7-driven vector (ampicillin resistance) behind His_6_ affinity tag and TEV (tobacco etch virus) protease cleavage site. After the cleavage, a cloning artifact of five amino acids (SNAAS) remained at the N-terminus of JASPer.

Chemically competent NiCo21 pRARE2 (DE3) *Escherichia coli* (NEB C2529H with additional plasmid coding rare codons and chloramphenicol resistance) were transformed by heat shock with JASPer plasmid and cultivated on LB agar medium (100 μg/ml ampicillin, 25 μg/ml chloramphenicol, and 1% glucose) overnight at 37°C. Colonies were resuspended in LB broth (Sigma) supplemented with 100 μg/ml ampicillin, 25 μg/ml chloramphenicol, and 0.5% glycerol to OD_600_= 0.05. Bacterial cultures were cultivated in LEX Epiphyte 3 bioreactor at 37°C until OD_600_ = 0.8 and then transferred to 18°C. Protein expression was induced with 400 μM isopropyl-β-d-thiogalactopyranoside. The cells were harvested after 20-h expression by centrifugation (5000 × *g*, 4°C, 20 min) and were stored at −80°C.

Bacterial cells were lysated by Emulsiflex C3 in lysis buffer (25 mM Tris–HCl, pH 7.5, 1 M NaCl, 2 mM β-mercaptoethanol, 10 μM EDTA) with protease inhibitor cocktail tablet (EDTA-free) and DNase I. The lysate was cleared by centrifugation (25 000 × *g*, 4°C, 30 min) and loaded on Ni-NTA resin (Sigma). The bound protein (with His_6_ tag) was then eluted by imidazole buffer (25 mM Tris–HCl, pH 7.5, 1 M NaCl, 2 mM β-mercaptoethanol, 250 mM imidazole). The purest fractions were dialyzed in the presence of TEV protease to lysis buffer overnight. JASPer without His_6_ tag was present in the flow-through fraction in the second Ni affinity chromatography. JASPer sample was concentrated using an Amicon Ultra centrifugal filtration unit and loaded onto size exclusion chromatography Superdex 200 10/300 GL in buffer (25 mM Tris–HCl, pH 7.5, 150 mM NaCl, 1 mM TCEP, 10 μM EDTA). The final concentration of the purified protein was determined on a NanoDrop spectrophotometer, and its purity was evaluated by SDS–PAGE stained by Coomassie Brilliant Blue.

Nucleosomes were prepared as described in [31].

### Microscale thermophoresis

JASPer protein (1–475) was dialyzed overnight to microscale thermophoresis (MST) buffer without Tween (20 mM HEPES, pH 7.5, 50 mM NaCl, 1 mM TCEP). The stock of exchanged protein was divided into equal aliquots and stored at −80°C. After thawing, each aliquot was centrifuged (20 000 × *g*, 4°C, 10 min), and the protein concentration was remeasured. JASPer at a constant concentration of 0.4 μM was mixed in 16-step dilution series (2:1 ratio, 0.5 nM and reaching up to 16 μM) with reconstituted nucleosomes (147 bp H3K36me0/2/3). The samples were adjusted to final volume of 30 μl MST buffer (20 mM HEPES, pH 7.5, 50 mM NaCl, 1 mM TCEP, 0.05% Tween 20). All reaction tubes were centrifuged and incubated for 15 min at RT. Mixtures were loaded into Monolith NT.LabelFree premium capillaries and measured at 20% LED power and 40% MST power on a NanoTemper Monolith NT.LabelFree device at 24°C. Each mixture was prepared twice, where each replicate was measured in technical triplicates. Values were fitted in the GraphPad Prism using a built-in equation for specific binding with Hill slope.

## Data analysis

### Read processing

ChIP-seq sequence reads were demultiplexed by JE demultiplexer [54] using the barcodes from the Illumina index read files. Demultiplexed files were aligned either to *Drosophila melanogaster* reference genome (BDGP6, release 104) or independently to *D. virilis* genome (droVir3, February 2006) using Bowtie2 [55] version 2.28.0 (parameter “--end-to-end --very-sensitive --no-unal --no-mixed --no-discordant -X 500 -I 10”) and filtered for quality using samtools 1.6 [56] with a MAPQ score cutoff of -q 10. For transposons, a custom genome containing repetitive regions was used as before [36] at the alignment step. Tag directories and input-normalized coverage files were generated using Homer [57] with the parameter -totalReads set to the number of reads mapped to *D. virilis* genome for spike-in normalization. Input-normalized, scaled *D. melanogaster* coverage per base pair files were visualized using the Integrative Genomics Viewer and were used for all analyses involving assessment of changes in ChIP signals (from Fig. 3) [58]. Replicate coverages were first analyzed independently to confirm similarities in HMT dependence patterns after which they were averaged for subsequent analyses. Resizing of coverages to fixed window sizes of mean signal was performed with bedops and bedmap [59].

Published RNA-seq reads were obtained from respective sources using sra prefetch and processed according to [36]. Single-end parameters were used for read alignment by STAR for [41] and paired-end settings for [51].

### ChIP-seq peak calling and annotation

Broad domains of modified H3K36me1/2/3, H327me3, and H3K9me2 were called using MACS2 v2.1.2 [60] bdgpeakcall function using parameters -l 1000 (-c 3 for K36me1/2/3; -c 0.8 for K27me3; -c 2.0 for K9me2). Manipulation (filtering, merging, etc.) of peak sets was performed with BEDTOOLS2 v2.28.0. Genomic annotation of peaks was done using HOMER annotatePeaks.pl script.

### Data analysis and plotting

Data analysis was conducted in R [61] using tidyverse libraries. ChromoMaps were generated using R chromoMap package [62]. Clustered heatmaps were made using R package “ComplexHeatmap” [63]. Chromatin state annotations were derived from modENCODE [2]. Gene annotations were obtained from FlyBase GTF annotations BDGP6 release 104. Only genes associated with a unique FlyBase (FBgn) ID (*N* = 17 800) were used for subsequent analyses. Detailed explanation for certain analyses can be found within provided R scripts.

### Differential binding analysis

To identify significant differential K36me1/2/3 regions across RNAi conditions, csaw v1.24.3 [64] was used. Reads for each unique experimental sample were counted into sliding windows of 250 bp across the entire genome. Windows were filtered using filterWindowsControl function using the corresponding input profiles as controls. To calculate normalization factors derived from the spike-in, normFactors function was applied on *D. virilis* bins containing high ChIP signal. These high-signal bins in the spike-in genome should not vary across the different *D. melanogaster* RNAi conditions; thus, any systematic differences in the signal across the libraries reflect technical biases that can be removed by normFactor scaling. Differential binding was assessed for using the quasi-likelihood framework in the edgeR package v3.32.1 with robust = TRUE for glmQLFit. The design matrix was constructed using a layout specifying the RNAi treatment as well as the experimental batch. Proximal tested windows were merged into regions of maximum 3 kb by clusterWindows with a cluster-level false discovery rate (FDR) target of 0.05.

### Data transformation

*Z*-score data transformation for average signal within 5-kb genomic windows (Fig. 3) was performed similarly to a previously described method [50]. Average feature coverage instead of RPKM was used in calculations. A consensus peak set representing all genomic regions containing at least one K36 modification in any RNAi condition was further used to filter windows before *Z*-score representation in Fig. 3. Proportional change in average gene signal used in Fig. 6 was defined as [(HMT RNAi − control RNAi)/control RNAi].

### External datasets

**Table:** 

Dataset	Source
Ash1 ChIP (S2 cells)	Huang *et al.* [41]
H3K36me2 ChIP in Ash1 RNAi (S2 cells)	Huang *et al.* [41]
RNA-seq in Ash1 RNAi (S2 cells)	Huang *et al.* [41]
Chromatin states	Kharchenko *et al.* [2]
H3K36me3 modENCODE (S2 cells)	modENCODE (GSE20785)
H3K36me3 native ChIP (fly)	Chaouch *et al.* [50]
H3K36me3-modified MNase ChIP (S2 cells)	Albig *et al.* [36]
Su (var) 3–7	modENCODE (GSE23487)
H3K9me2	modENCODE (GSE20792)
H4K20me1	modENCODE (GSE27743)
H3K27ac	modENCODE (GSE20779)
H3K4me1	modENCODE (GSE32826)
Nurf	modENCODE (GSE20829)
H2B-ub	modENCODE (GSE20773)
H3K79me3	modENCODE (GSE45090)
Kdm4A	modENCODE (GSE32839)
Kdm2	modENCODE (GSE45061)
MRG15	modENCODE (GSE25367)
Beaf-32	modENCODE (GSE20760)
SMC3	modENCODE (GSE45054)
CTCF	modENCODE (GSE32750)
H3K27me3	modENCODE (GSE20781)
H3.3	Henikoff *et al.* [65]
SNR1	Hendy *et al.* [66]
ISWI	Hendy *et al.* [66]
RNA-seq in NSD/Set2 RNAi (S2 cells)	Depierre *et al.* [51]
WT S2 cell RNA-seq	Albig *et al.* [36]
Fly HMT KO RNA-seq	Lindehell *et al.* [28]
FlyAtlas tissue expression data	Chintapalli *et al.* [67]
H3K36me2/3 in HMT KO (C3H10T cells)	Weinberg *et al.* [13]
RNA-seq of WT C3H10T cells	Weinberg *et al.* [13]
H3K36me3 in HMT dTAG cells (mESC)	Sun *et al.* [68]

Genomic coordinates of datasets previously aligned to genome version dm3 were transformed to newer dm6 using liftOver tool [69].

## Results

### H3K36 methylation states and methyltransferases mark distinct chromatin types in S2 cells

To study the genomic distribution of the histone H3K36 methylation states (and their corresponding HMTs), we generated high-resolution MNase chromatin immunoprecipitation (ChIP-seq) profiles in male *Drosophila* S2 cells. Our optimized protocol includes the solubilization of chromatin through mild shearing, yielding significantly more signals at mappable heterochromatin compared to other methods (Supplementary Fig. S1A).

**Figure 1.**
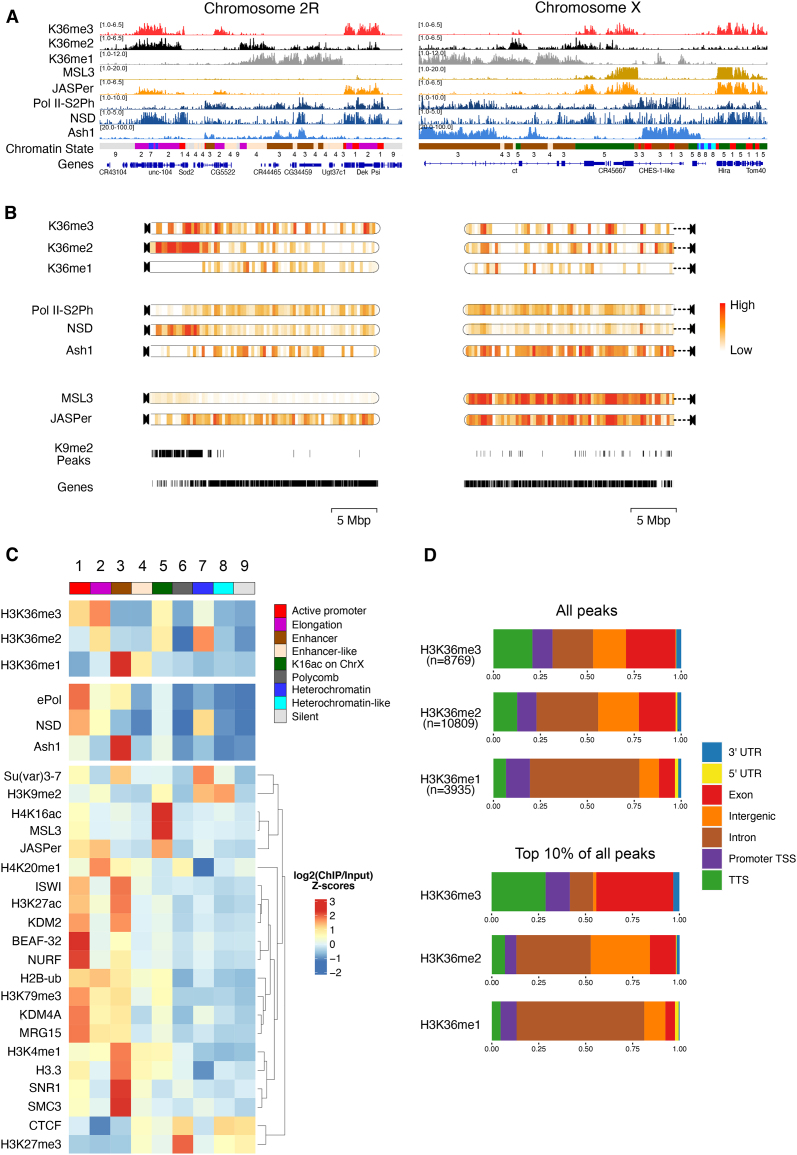
H3K36 modifications and HMTs mark distinct chromatin states in male *Drosophila* cells. (**A**) Genome browser profiles for representative *Drosophila* chromosomes 2R and X MNase ChIP of K36me1/2/3, the K36me3 reader proteins JASPer and MSL3, and the HMTs NSD and Ash1. “ePol” refers to the signal generated by an antibody against RBP1-S2ph, as a proxy for RNA-polymerase-S2ph-interacting Set2. The Ash1 profile was taken from [41]. The nine-state ChromHMM (modENCODE) is color-coded and explained in panel (C). (**B**) ChromoMaps representing steady-state enrichment of K36me1/2/3, HMTs, and K36me3 readers in 10-kb genomic bins for chromosomes 2R and X. Scale bars are different for each chromoMap to facilitate visualization of changes. The individual scale values for each state and chromosome are shown in Supplementary Fig. S1. Tracks representing H3K9me2 peaks and annotated genes serve as a reference for mappable PCH domains and transcribed chromatin, respectively. (**C**) Chromatin state enrichment (nine-state ChromHMM [2]) for K36me1/2/3, HMTs, and K36me3 readers. Published ChIP-seq/CUT&RUN profiles of histone modifications and other chromatin proteins were hierarchically clustered to highlight differences between chromatin states. (**D**) Genomic features either marked by all K36me1/2/3 domains (top) or filtered for the strongest 10% bottom.

We mapped K36me1/2/3 using highly specific antibodies [70], the HMT NSD and the elongating RNA polymerase II (ePol) as a proxy for Set2 (for which no ChIP-grade antibodies exist). A recently published Ash1 ChIP profile [41] was included. We also mapped two *bona fide* K36me3 binders: JASPer, a PWWP-domain reader that colocalizes with K36me3 genome-wide, and MSL3, a K36me3 chromodomain reader restricted to the X chromosome [36]. The browser views (Fig. 1A) reveal the distinct distributions of considered features, in particular for K36me1/2 and K36me3. To visualize the differences in the distribution of the K36 methylation states as well as the associated factors at a chromosome-wide level, we generated chromoMaps representing average ChIP signals in 10-kb bins superimposed on the chromosomes (Fig. 1B and Supplementary Fig. S1B). Tracks depicting annotated genes as well as K9me2 peaks highlighting the gene-poor mappable pericentric heterochromatin (PCH) regions were included for reference. Of note, the PCH region is not prominently represented on the X chromosome because it is poorly mapped in the dm6 reference genome. K36me1 is largely euchromatic, and only partly overlaps with K36me2/3. K36me2 is heterochromatic, but also present in stretches of euchromatin. Surprisingly, K36me3 is distributed rather uniformly between euchromatin and PCH, where it is not restricted to genes (Supplementary Fig. S1A). Set2 (inferred from ePol) and Ash1 are largely enriched at euchromatin, while NSD showed a clear heterochromatin enrichment but is also found in euchromatic regions. The two K36me3 reader proteins JASPer and MSL3 correlate best with K36me3 as already described (Fig. 1A). Of note, the chromoMaps of the X chromosome for MSL3 and JASPer predominantly illustrate the X-chromosomal enrichment due to the association with dosage compensation.

We called peaks to estimate the proportion of genome decorated by each K36me mark. K36me2 and K36me3 occupy a similar proportion of the genome (∼20%), while K36me1 is concentrated more locally (∼12%). Correlating the coverage to estimated absolute modification numbers derived from late-stage embryos [71] suggests that K36me1 is the most densely placed mark, followed by K36me2 and then K36me3 (Supplementary Fig. S1C).

To examine the distribution of the marks and the associated factors at a higher resolution, we next correlated ChIP enrichments to each of the nine functional chromatin states as defined by the modENCODE consortium (Fig. 1C). We included various modENCODE ChIP datasets as well as more recently published profiles generated from published data to highlight features distinguishing the nine states. This revealed several interesting patterns confirming the chromoMap observations. K36me3 is strongly enriched within euchromatic states 1 and 2 representing active promoters and transcribed chromatin, but also shows a mild heterochromatin enrichment (state 7). K36me2 marks similar states but is much more abundant at heterochromatin. In contrast, K36me1 is almost exclusively present at enhancer-like chromatin states 3 and 4.

Correlating the HMT occupancies to the different K36 methylation states showed that Ash1 is most strongly enriched with K36me1 at enhancers (state 3). NSD is distributed between eu- and heterochromatin (states 1 and 7). Set2 (ePol) is rather restricted to euchromatin at transcribed regions (states 1 and 2) and enhancers (state 3) but rather not correlating with the mild K36me3 enrichment in heterochromatin (state 7). The dual distribution of NSD was confirmed by immunofluorescence confocal microscopy (Supplementary Fig. S1D). In addition to a diffuse signal in euchromatin, intense speckles adjacent to (but not overlapping) DAPI-dense chromocenters were observed. Both populations were sensitive to RNAi against NSD, confirming the specificity of the antibody.

We then intersected the peaks of K36me1/2/3 with genomic features considering the strongest peaks (top 10%) to highlight differences (Fig. 1D). K36me1 is strongly enriched in introns, in agreement with its localization to enhancers. Strong K36me3 signals accumulate at TTS and exons, whereas K36me2 marks mostly genic features in particular introns but also some intergenic regions. In conclusion, different H3K36 methylation states label distinct types of chromatin and genomic features. NSD stands out due to its association with both eu- and heterochromatin.

### Contribution of methyltransferases to different H3K36 methylation states

To determine the contribution of HMTs to the three K36 methylation states, we depleted each HMT individually as well as in combination by RNAi and followed the levels of the HMTs and K36me1/2/3 by quantitative western blotting. The depletion of Set2 and NSD was efficient (>90% and ∼80%, respectively, Supplementary Fig. S2A). Because the Ash1 antibody performed poorly in western blotting, we estimated Ash1 mRNA levels by RT-qPCR too, which revealed a 75% reduction upon RNAi (Supplementary Fig. S2B).

**Figure 2.**
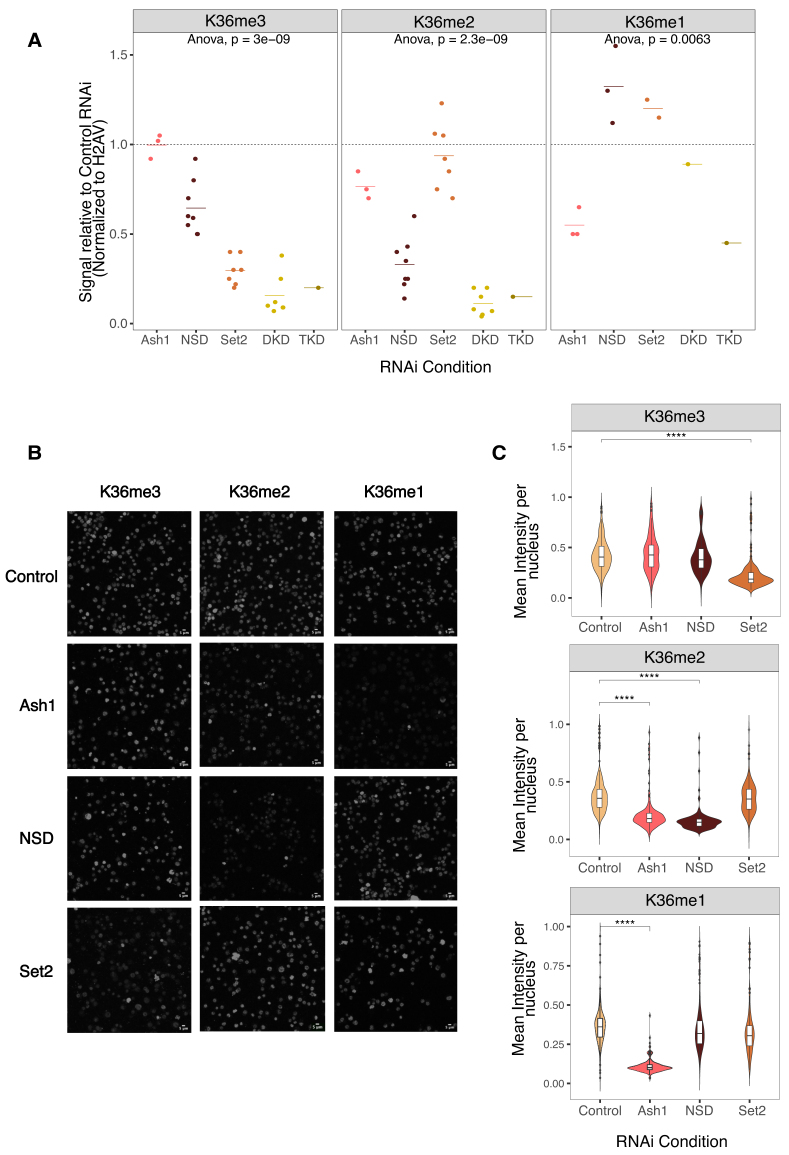
Effect of HMT depletion on H3K36 methylation bulk levels reveals distinct HMT dependences in male cells. (**A**) K36me1/2/3 levels in whole cell extracts from S2 cells were determined by quantitative western blotting using specific antibodies. Cells were treated with RNAi against Ash1, NSD, Set2, NSD + Set2 (DKD), or NSD + Set2 + Ash1 (TKD). An irrelevant GST RNAi served as control. Representative blots are shown in Supplementary Fig. S2C. Source data for all blots can be found in Zenodo repository (see the “Data availability” section). Values were normalized to histone H2AV signals on the same membrane and are represented as fraction relative to GST RNAi, which was run on same blot. Each dot represents an independent biological replicate. Calculated ANOVA *P*-values (null hypothesis: difference between means = 0) are presented for each antibody. (**B**) Representative IFM images for α-H3K36me1/2/3 in S2 cells treated with RNAi against GST (control), Ash1, NSD, or Set2. The scale bar is 5 μm. (**C**) Quantification of IFM images [*n* ∼ 500 nuclei from first biological replicate, shown in panel (B)]. ANOVA followed by post-hoc Tukey HSD was performed to identify groups with significantly different mean relative to GST RNAi (****P < .001).

We first followed bulk changes in K36 methylation upon HMT RNAi (Fig. 2A, representative blots in Supplementary Fig. S2C). Loss of Set2 led to ∼75% reduction of western blot signal for K36me3, while K36me2 appeared unaffected. Depletion of NSD strongly reduced western blot signal for K36me2 (∼80%), but led to only modest reduction of K36me3 signal (∼40%) in agreement with previous reports [25, 43]. These observations are at odds with the idea that NSD only generates intermediates for Set2-dependent trimethylation. We also observed similar trends in female Kc cells, ruling out cell- or sex-specific effects (Supplementary Fig. S2D and E).

Interestingly, K36me1 was rather unaffected in RNAi of Set2 and/or NSD, arguing against the idea that NSD is the major K36me1 methyltransferase. Partial depletion of Ash1 did not affect K36me3 levels, moderately decreased K36me2 by ∼20%, but led to very prominent reduction of K36me1 (50%) as indicated by their corresponding western blot signals. This reduction may be an underestimation, since the previously mentioned modENCODE study [70] reported that certain lots of K36me1 antibody have mild reactivity toward unmodified K36 in western blot assays.

We confirmed the western blot trends by IFM (Fig. 2B), quantifying the K36me1/2/3 signals upon HMT depletion (Fig. 2C and Supplementary Fig. S2F). K36me1 was exclusively decreased upon Ash1 RNAi. K36me2 was decreased reproducibly only in NSD RNAi, while an Ash1-dependent decrease was observed in only one replicate (Supplementary Fig. S2F). K36me3 was reduced in a Set2-dependent manner. The immunofluorescence staining did not reflect the reduction in K36me3 upon NSD RNAi found in western blot, possibly due to the sensitivity limit on one hand and the local distribution of the changes on the other hand (see below).

These results suggest that K36me1/2 do not simply serve as intermediates of trimethylation. They also reveal a complex interplay between the three HMTs, where each individual enzyme predominantly catalyzes one K36 methylation state, and only partially contributes to the others. Given the different distributions of K36me1/2/3 and HMTs genome-wide (Fig. 1A–C), the differences may also be restricted to some genomic locations. We thus expect different phenotypes upon HMT loss-of-function mutants in flies. To explore this possibility, we reanalyzed published transcriptome data of NSD, Set2, and Ash1 knockout mutant fly brain [28], focusing on gene-level correlations. Indeed, the significantly deregulated genes poorly correlate between individual HMT mutants (Supplementary Fig. S3), especially between Set2 and NSD, in support of the hypothesis of distinct roles of Set2 and NSD in gene regulation.

### Chromosome locus-specific H3K36 methylation changes upon HMT depletions

**Figure 3.**
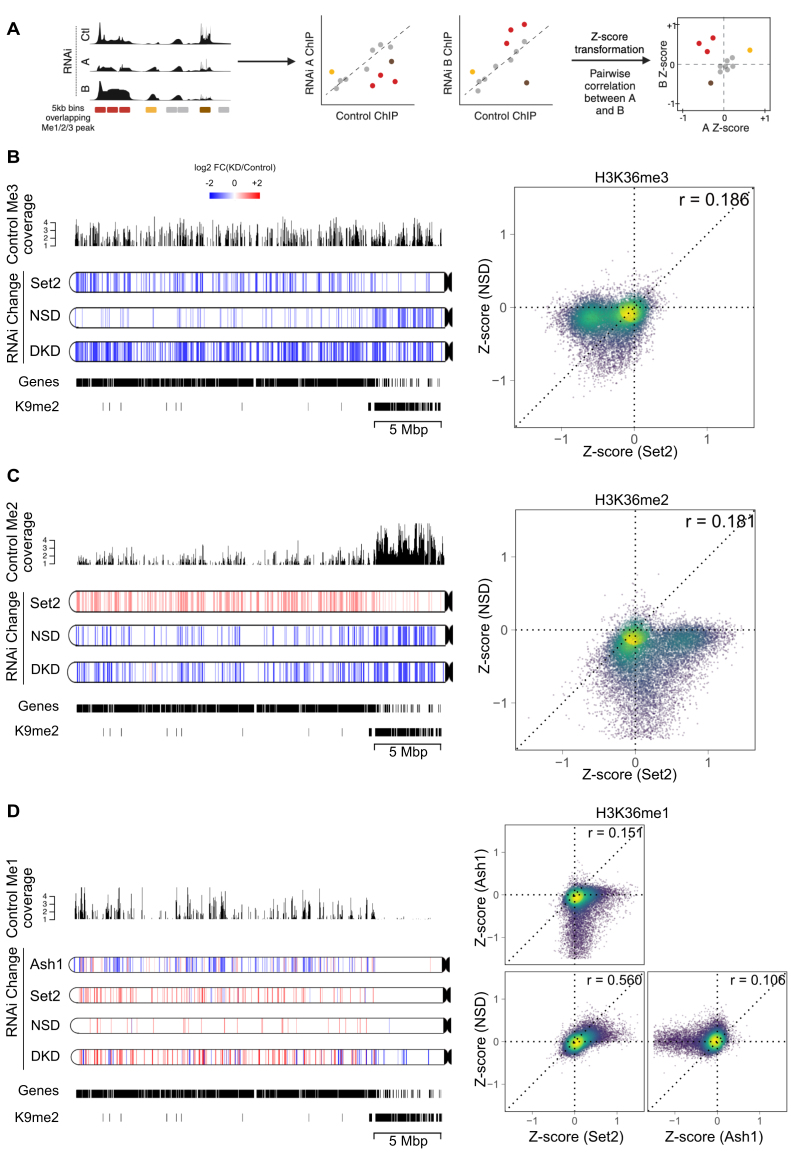
HMTs act on largely distinct genomic regions. (**A**) Schematic depicting *Z*-score analysis workflow. Pairwise *Z*-score scatter plots representing correlations among changes in ChIP signal upon RNAi of HMTs with respect to control RNAi in genome-wide 5-kb bins. Only bins overlapping at least one of K36me1/2/3 peaks in any RNAi condition were included. A negative *Z*-score indicates a reduction relative to control, while a positive *Z*-score indicates an increase. Schematic was generated with BioRender.com. (**B**–**D**) Pairwise *Z*-score scatter plots for K36me1/2/3 upon RNAi of indicated factor (right). The color overlaid on the scatter indicates the local density of points. Pearson correlations are provided for each pair. Corresponding chromoMaps representing regions of significant difference in K36me3/2/1 signal for indicated RNAi conditions as derived from csaw analysis for chromosome 3L at 2-kb resolution (left). The color of the regions (as indicated by the common scale in Fig. 3B) represents log_2_-transformed value of number of normalized reads in RNAi condition relative to control condition. Control K36me3/2/1 signal overlaid above chromoMaps aids interpretation of relative changes. K9me2 peaks and gene annotations are provided for reference.

We next explored the genome-wide changes in K36 methylation after depletion of HMTs (Fig. 3). *Z*-scores representing the direction and magnitude of change in K36me ChIP signal within 5-kb genomic bins were calculated for each HMT RNAi relative to the control (Fig. 3A). To identify genomic regions that show significantly altered K36me1/2/3 upon RNAi, we adopted a statistical approach using the csaw pipeline (see the “Materials and methods” section). The differentially marked regions (FDR < 0.05; at least 50% loss or gain of signal in control RNAi) obtained from this analysis were further visualized using high-resolution 2-kb chromoMaps to provide information regarding spatial patterns of methylation changes in each RNAi condition. For conciseness, only chromosome 3L is shown (all chromosomes are shown in Supplementary Fig. S4A–C). Profiles representing the respective K36me levels in unperturbed S2 cells above each chromoMap aid the interpretation of the relative changes, while the gene annotation and H3K9me2 peak tracks illustrate local chromatin environment (Fig. 3B–D).

The chromoMaps for K36me3 show that depletion of Set2 leads to a strong reduction of predominantly euchromatic K36me3 as previously described [35, 37], while depletion of NSD leads to loss of K36me3 at gene-poor PCH. This general distinction is not absolute: a subset of euchromatic bins (possibly distinct from Set2-dependent regions as well as euchromatic K9me2 nanodomains) show NSD dependence, while a few heterochromatic bins were identified as significantly decreasing upon Set2 knockdown (Fig. 3B).

To directly compare the differential effect of HMT depletion at higher resolution, we visualized pairwise correlations at genomic bins that contain at least one K36 modification as *Z*-score scatter plots (see principle in Fig. 3A). Off-center dots indicate the bins affected by Set2 or NSD or by both depletions (with points lying on the diagonal line representing bins equally affected by both), while bins centered around (0, 0) represent unchanging bins, which are typically “background” regions of low K36me3 signal (but are likely decorated by K36me1 or K36me2) (Fig. 3B and C). K36me3 is reduced in a large fraction of genomic regions upon depletion of Set2 only (bins along the horizontal line), consistent with its role as a major trimethyltransferase. On the other hand, NSD RNAi leads to strong reduction of K36me3 in certain bins that only show partial sensitivity to Set2 RNAi. These observations indicate that Set2 predominantly mediates euchromatic K36me3 independently of NSD, and minimally contributes to heterochromatic K36me3, which is mostly dependent on NSD.

The contributions of NSD and Set2 to K36me2 are more intricate (Fig. 3C), likely due to the dual pathways involved: methylation (me0/1 → me2) and demethylation (me3 → me2) reactions. The chromoMaps illustrate that K36me2 at heterochromatin and at certain euchromatic bins is dependent on NSD. Interestingly, many bins lose both H3K36me2 and H3K36me3 upon NSD RNAi (compare Fig. 3B and C). Conversely, K36me2 at heterochromatin is largely unaffected upon Set2 RNAi but strongly decreased upon NSD RNAi. The bins where K36me2 is reduced in an NSD-dependent manner are in the bottom half of the scatter plot (Fig. 3C).

Remarkably, euchromatic K36me2 levels are clearly increased upon depletion of Set2 mostly within bins that are accompanied by corresponding drops in K36me3 levels and were previously assumed to be the product of demethylation of K36me3 (compare Set2 RNAi in Fig. 3B and C). The corresponding bins are represented along the horizontal line on the right side of the corresponding scatter plot suggesting that they are not sensitive to NSD depletion (Fig. 3C). Interestingly, this strong increase observed upon Set2 RNAi is lost in the DKD chromoMaps, suggesting that it is NSD dependent and not only arising by demethylation of me3 → me2. Conceivably, the dimethylation activity of NSD at euchromatin is augmented in the absence of Set2. We ensured that this effect was not due to a systematic bias in library preparation by verifying ChIP signals at several diagnostic loci (data not shown).

Lastly, chromoMaps and scatter plots highlight a strong loss of K36me1 upon depletion of Ash1 at loci with a strong enrichment of K36me1 in control cells confirming that Ash1 is the major K36me1 transferase in S2 cells (Fig. 3D). Depletions of Set2 or NSD are rather associated with an increased K36me1 in distinct bins that have very little K36me1 in control cells, and likely result from demethylation of K36me2/3.

Taken together, our results suggest that the three methyltransferases establish the genomic K36 methylation signatures according to locus-specific rules. Each HMT primarily establishes the methylation state in distinct domains and may contribute to a lesser extent in other domains. In addition, the strong NSD-dependent K36me2 increase at euchromatin in the absence of Set2 suggests an indirect activation of NSD beyond its primary designated domains.

### A gene-centric view of the K36 methylation landscape

**Figure 4.**
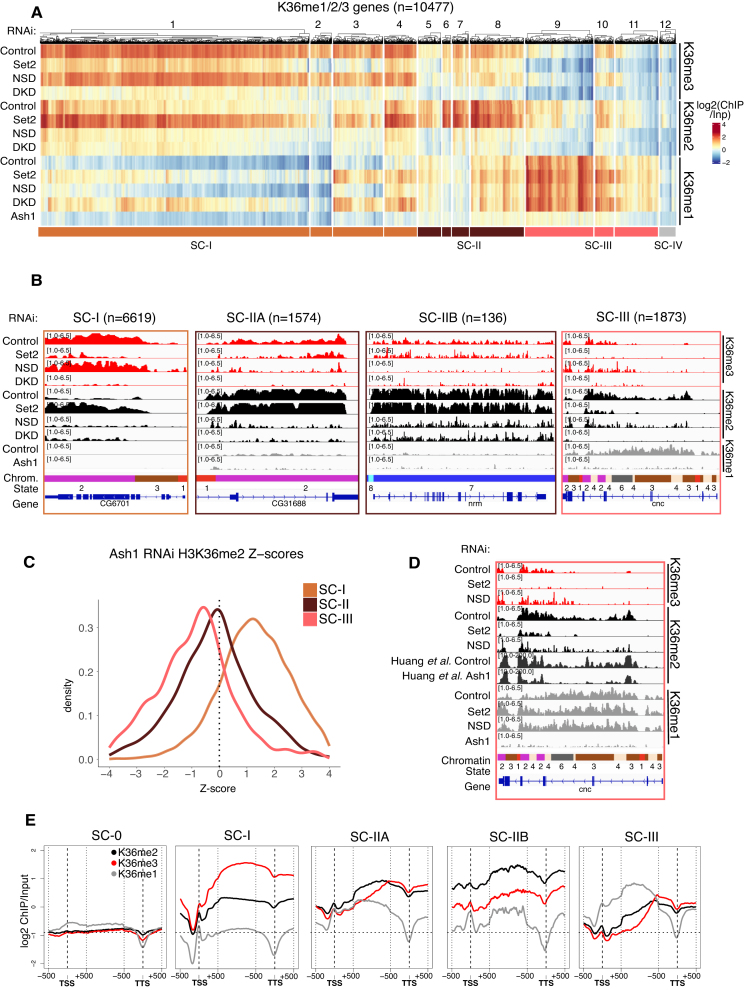
A gene-centric view of the K36 methylation landscape. (**A**) Clustered heatmaps of gene body-averaged ChIP signal for K36me1/2/3 indicated on the right and RNAi condition indicated on the left. Only genes overlapping at least one of K36me1/2/3 peaks in any RNAi condition (*n* = 10 477) were used for clustering. Clusters are numbered 1–12 as indicated above the heatmap. The track below the heatmap represents the manual grouping of clusters to define superclusters that display similar patterns, resulting in SC-I (*n* = 6619), SC-II (*n* = 1710), and SC-III (*n* = 1873). (**B**) Genome browser profiles of representative genes from each supercluster along with the number of grouped genes. Supercluster II was further classified into euchromatic (IIA) or heterochromatic (IIB) based on overlap with H3K9me2 peaks. RNAi condition and immunoprecipitation target indicated on the left; nine-state chromatin states (as in Fig. 1C) serve as a reference. Representative genes shown for SC-I, SC-IIA, SC-IIB, and SC-III lie within clusters 1, 8, 7, and 9, respectively. (**C**) Density plot of *Z*-scores representing change in K36me2 signal upon Ash1 RNAi [41] for superclusters defined in Fig. 4A. (**D**) Genome browser profiles of supercluster 3 representative gene highlighting effect of Ash1 RNAi on K36me2. RNAi condition and immunoprecipitation target indicated on the left; chromatin states (as in Fig. 1C) shown for reference. (**E**) Cumulative plots for each cluster representing gene body relative distributions of K36me1/2/3. 1-kb regions centered around TSS and TTS are unscaled, while the rest of the gene body was scaled to 500 bins. SC-0 genes lack any detectable H3K36 methylation and serve as a reference for zero signal/baseline. This is also represented by the horizontal dotted line in all individual plots. Only genes of minimum length of 1500 bp were included in the analysis.

The observation of distinct contributions of the three relevant HMTs to locus-specific methylation signatures prompted a more gene-centric analysis. K36 methylations are predominantly found on transcribed chromatin (Fig. 1D). We selected around 10 500 genes enriched for at least one K36 methylation state in any one condition. The signals over the gene bodies were averaged and the resulting profiles clustered across all RNAi conditions. This yielded 12 clusters that once again illustrate the complexity of dynamic K36 methylation patterns across the genome. To facilitate a first interpretation of broad biological patterns while avoiding overinterpretation, we manually merged the clusters into three superclusters (supercluster I = clusters 1–4; supercluster II = clusters 5–8; supercluster III = clusters 9–11; Fig. 4A). The minor supercluster IV (cluster 12, gray) contains a few hundred genes with very low K36me signals and was excluded from all subsequent analyses. The genes within the superclusters I–III are strongly correlated to specific K36 methylation profiles in steady state and tend to show consistent changes upon HMT depletion. Genome browser profiles for representative genes in each supercluster are shown in Fig. 4B. Interestingly, these clusters correlate with distinct chromatin factors that may provide hints about their function/regulation (Supplementary Fig. S5A).

#### Supercluster I genes depend on Set2 for K36 methylation

Supercluster I consists of genes that have the highest average K36me3 signal and very low me1/2 in control conditions exemplified by the gene CG6701 (Fig. 4A and B). At these genes, K36me3 largely depends on Set2. The reduction in K36me3 in the absence of Set2 is accompanied by an increase in K36me2 and, most prominently in clusters 3 and 4, an increase in K36me1. Variable effects may be due to heterogeneity in locus-specific histone demethylase activity (see below and Fig. 6). We also observe a mild general decrease in K36me2 upon depletion of NSD (Fig. 4A), confirming that NSD weakly contributes to dimethylation at some genes within this supercluster.

The patterns observed upon combined depletion of both Set2 and NSD (DKD) differ from those upon Set2 KD alone, allowing to distinguish simple additive effects from more complex relationships. For example, many genes show a slightly stronger reduction of K36me3 in DKD compared to Set2 knockdown alone, pointing at a mild additive contribution of NSD to K36me3. Furthermore, as also observed genome-wide, the pronounced increase in K36me2 (but not the mild increase of K36me1) observed upon Set2 RNAi is absent in the DKD condition, arguing for an ectopic activation of NSD in the absence of Set2.

Altogether, at SC-I genes Set2 generates K36me3 with very little intermediate K36me1/2 and does mostly not rely on prior methylation by NSD.

#### NSD is responsible for H3K36me2/3 at euchromatic and heterochromatic genes in supercluster II

Supercluster II consists of 1710 genes with high K36me2, moderate K36me3, and variable K36me1 levels in unperturbed cells. Only a minor fraction of these genes (136) are located in PCH and carry the heterochromatic K9me2 mark (Fig. 4B and Supplementary Fig. S6A). The abundant K36me2 is strongly reduced upon depletion of NSD, and is mostly unaffected upon depletion of Set2. This reduction of K36me2 at SC-II is often accompanied by a moderate reduction in K36me3 at sites where also Set2 is active. Knocking down both methylases reveals additive contributions of Set2 and NSD toward K36me3 at this supercluster. This suggests that at those genes NSD can trimethylate H3K36 in addition to Set2. Evidently, NSD is essential for K36me2/3 in this cluster of genes, of which 90% reside in euchromatin. The low levels of K36me1 observed in a subset of the clusters are mostly sensitive to Ash1 RNAi only. For further downstream analyses (see below), we distinguish euchromatic genes (supercluster SC-IIA) from heterochromatic NSD-dependent genes (supercluster SC-IIB) for clarity.

#### Ash1 is responsible for H3K36me1 in supercluster III

Supercluster III genes stand out because they are strongly marked by K36me1, which is markedly reduced by depletion of Ash1, but not affected by either Set2 or NSD depletion. This confirms that K36me1 is a state in itself and not only a methylation intermediate (Fig. 4A).

A subset of supercluster III genes are decorated by low to moderate levels of K36me2 and K36me3. At these genes, K36me3 appears to be particularly Set2 dependent, while K36me2 is NSD dependent. We were intrigued by the NSD dependence of K36me2 at these genes, given that Ash1 has also been previously described as dimethyltransferase [41]. We used a previously published dataset [41] to show that K36me2 at genes within the supercluster III indeed additionally depends on Ash1 (Fig. 4C and D). The data are compatible with a scenario, in which NSD further methylates Ash1-dependent K36me1 in this context. Remarkably, the above-mentioned “indirect activation” of NSD activity upon Set2 depletion is also observed in response to Ash1 RNAi at SC-I genes and not SC-II genes (Fig. 4C). Evidently, the NSD-dependent dimethylation at SC-I appears to be strongly increased whenever either Set2 or Ash1 is depleted, suggesting a rather locus-specific phenomenon (see the “Discussion” section).

#### Context-dependent distribution of H3K36 methylation states along gene bodies

H3K36me3 is frequently referred to as a histone mark enriched toward 3′ ends of genes. Anecdotal genome browser observations (Fig. 4B) suggest that the intragenic location of K36 methylation states differs. To investigate this more systematically, we generated cumulative profiles of K36 methylation states along scaled gene bodies between transcription start sites (TSS) and transcription termination sites (TTS), separately for each supercluster (Fig. 4E). Unmethylated genes (SC-0) that were not included in the clustering serve as an internal negative control.

Genes in supercluster I show a strong 3′ bias for K36me3 but not K36me2, consistent with previous reports [72]. K36me2 is broadly distributed throughout the bodies of the euchromatic genes in SC-IIA, whereas K36me1 drops toward the TTS and K36me3 increases sharply toward their 3′ ends. Such a modulation of K36me3 was not observed for the heterochromatic cluster II genes (SC-IIB), where the gene body signals are similar to those upstream of TSS and downstream of TTS. This observation fits the genome browser views (Supplementary Fig. S1A) that show heterochromatic genes embedded in larger K36me2/3 domains.

Finally, at supercluster III genes, K36me1 broadly marks the gene bodies, whereas K36me2/3 are enriched toward the 3′ ends of transcription units. Notably, the intragenic distribution of the dimethyl mark is more similar to K36me3 rather than K36me1, further supporting the idea that most K36me2/3 at these genes is deposited by NSD and Set2, respectively, and not directly by Ash1 (Fig. 4A).

The observation of distinct profiles of K36 methylation along transcription unit supports the concept of context-dependent methylation pathways.

### Genes of superclusters I, II, and III are enriched in ePol, NSD, and Ash1, respectively

**Figure 5.**
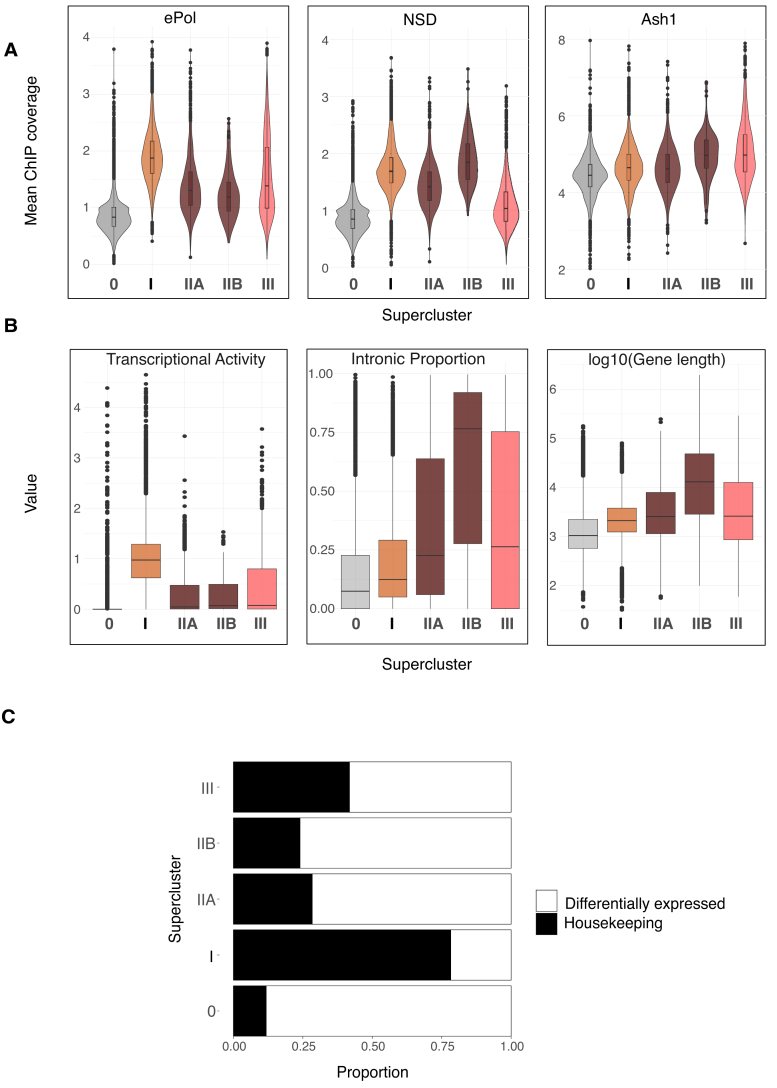
Gene clusters defined by HMT methylation patterns are correlated to different genic features. (**A**) Violin plots representing average ChIP signal for 2-kb windows around HMT gene body peaks for NSD, ePol, and Ash1 within superclusters defined in Fig. 4A. Supercluster 0 represents randomly sampled genes (*n* = 3000), which lack any detectable K36 methylation, and serves as reference for zero signal/baseline. (**B**) Boxplots showing the proportion of introns (sum of length of introns/total gene length), transcriptional activity (denoted by log_10_-transformed RNA-seq TPM values), and log_10_(gene length) for gene superclusters. (**C**) Proportion of tissue-specific/invariant genes for gene superclusters based on FlyAtlas expression data for 25 tissues.

The distinct distributions of K36 methylation marks at genes may be explained by targeting of the corresponding HMTs. If that was the case, we should find the HMT enriched at sites where K36 methylation is sensitive to its depletion. HMTs are often found enriched at distinct loci within larger domains of K36 methylation. To avoid averaging HMT ChIP coverage over the entire gene body, we selected the binding maxima for each HMT and calculated average signals within 2-kb windows around these maxima. The intensity distribution of the HMTs within the superclusters was visualized as violin plots (Fig. 5A). A set of 3000 randomly sampled unmethylated genes (SC-0) served as an internal negative control.

Set2 (defined by the presence of ePol) was strongly enriched on genes with Set2-dependent K36me2/3, most prominently supercluster I genes, followed by supercluster III genes (mainly contributed by cluster 10), which show a Set2-dependent K36me3 and an NSD-dependent K36me2. Expectedly, NSD enrichment was stronger at heterochromatic genes (SC-IIB) compared to NSD-dependent euchromatic genes (SC-IIA). NSD was also weakly enriched at supercluster III genes. Set2-dependent supercluster I genes showed comparable levels of NSD to SC-IIA genes suggesting that NSD may be recruited to SC-I genes although its contribution to K36me at those genes is subtle in control conditions but increased after Set2 depletion. Lastly, analyzing the published Ash1 profile [41], we confirm that Ash1 was enriched the most in supercluster III. Mild enrichment within superclusters I and II-A may be due to overlapping domains (Fig. 1A). The Ash1 enrichment observed within SC-IIB heterochromatic genes is probably contributed by nonspecific signals that remain in Ash1 RNAi condition (data not shown).

Overall, these observations suggest that the three HMTs are the dominant drivers of K36 methylation at their highlighted gene clusters, but may also contribute to K36 methylation in other contexts.

### Housekeeping functions are marked by Set2, while NSD and Ash1 target differentially expressed genes

The context for K36 methylation signatures (Fig. 4E) may relate to gene-specific features such as transcriptional activity, length, and intron content. We visualized those features for genes in each supercluster as boxplots (Fig. 5B). Genes in supercluster I were most highly transcribed and transcription activity correlated strongly with occupancy of the elongating RNA polymerase II (ePol) (Fig. 5A, left). Genes in superclusters II and III are less expressed in agreement with lower ePol occupancy, tend to be longer, and contain more introns. Given the specific patterns of steady-state transcription in the different superclusters, we explored whether perturbation of individual HMTs leads to altered transcription within their respective superclusters (Supplementary Fig. S7A–E). Interestingly, the majority of the genes do not change in expression upon RNAi of the corresponding HMT, suggesting that K36 methylation does not have a major influence on bulk transcript levels. Supplementary Fig. S7 presents a reanalysis and discussion of published transcriptome data.

Our observations show that genes of superclusters II and III are frequently long and intron-rich. Genes of this type are often differentially expressed across cell types, in contrast to housekeeping genes. To evaluate the degree of differential gene expression, we classified genes according to their FlyAtlas expression profiles for 25 different tissues [67], as either “tissue-invariant” or “differentially expressed” (Supplementary Fig. S8A), and calculated the proportion of these two classes across the superclusters. This revealed that genes in superclusters II and III were predominantly differentially expressed, while supercluster I genes were ubiquitously transcribed (“housekeeping genes”) (Fig. 5C). Tissue-specific genes are frequently subject to silencing by the Polycomb complex. Since K36 methylation has been linked to counteracting Polycomb-mediated K27me3 [41, 51], we explored whether the K27me3 landscape is perturbed upon individual K36 HMT RNAi (Supplementary Fig. S9A–F). While the majority of K27me3 domains remain unchanged upon HMT RNAi, we do observe several *de novo* K27me3 domains appearing within genes that lose K36 methylation bolstering the idea that K36 methylation contributes to restricting K27me3 spreading.

A further determinant of HMT targeting may relate to intron/exon content. K36me3 maps predominantly to exons, perhaps due to a slowing of ePol (and associated Set2) during splicing [4]. Other studies also documented K36 methylation on introns [3]. Anecdotal genome browser views (Fig. 4B) hinted that K36me1/2/3 may mark introns and exons differentially in the different superclusters. We therefore examined the exon/intron distribution of steady-state K36 methylation (Supplementary Fig. S10A). We found K36me3, but not K36me2, strongly enriched in exons versus introns in Set2-dependent supercluster I genes. Likewise, there is substantial exon bias for K36me3 but lesser bias for K36me2 at NSD-dependent SC-IIA genes. Because this exon bias at SC-IIA genes is not lost upon Set2 depletion (Supplementary Fig. S10B), we speculate that NSD may have a K36me3 exon bias as well, despite no known interaction with ePol. Since a much weaker bias is observed for SC-IIB genes, exon selection is not an intrinsic feature of NSD but rather depends on the chromatin context (Supplementary Fig. S10A). Supplementary Fig. S10B shows how the exon/intron distribution and bias shift upon depletion of the HMTs and reveals complex patterns that may be explored through future analyses.

The division of labor between HMTs for establishment of K36 methylation patterns genome-wide and the correlation to various genic features appear to be conserved during evolution. Similar SETD2-dependent and NSD1/2-dependent gene clusters were observed in two independent mouse datasets (Supplementary Fig. S11A).

### K36 methylation at transposable elements

Some transposable elements (TEs) are marked by H3K36me3 in *Drosophila* [36], and K36 HMTs were suggested to regulate TE expression [73]. We mapped K36me2/3 at various repeat families across RNAi conditions and clustered the profiles (Supplementary Fig. S12A). Roughly 50% of TE families were enriched in H3K36me2 or H3K36me3 in control condition. Most methylated transposon families have a high K36me2/low K36me3 and K36me3 is mostly dependent on Set2, while K36me2 is mostly dependent on NSD. These patterns are consistent with the fact that most copies of the TEs are embedded in PCH [74] and contribute to the bins highlighted in the chromoMaps of Fig. 3. Telomeric TEs and a few others were defined by rather high K36me3/low K36me2, which may be a consequence of higher transcription levels.

### H3K36me reader binding is determined by density and turnover of K36me3 and K36me2

**Figure 6.**
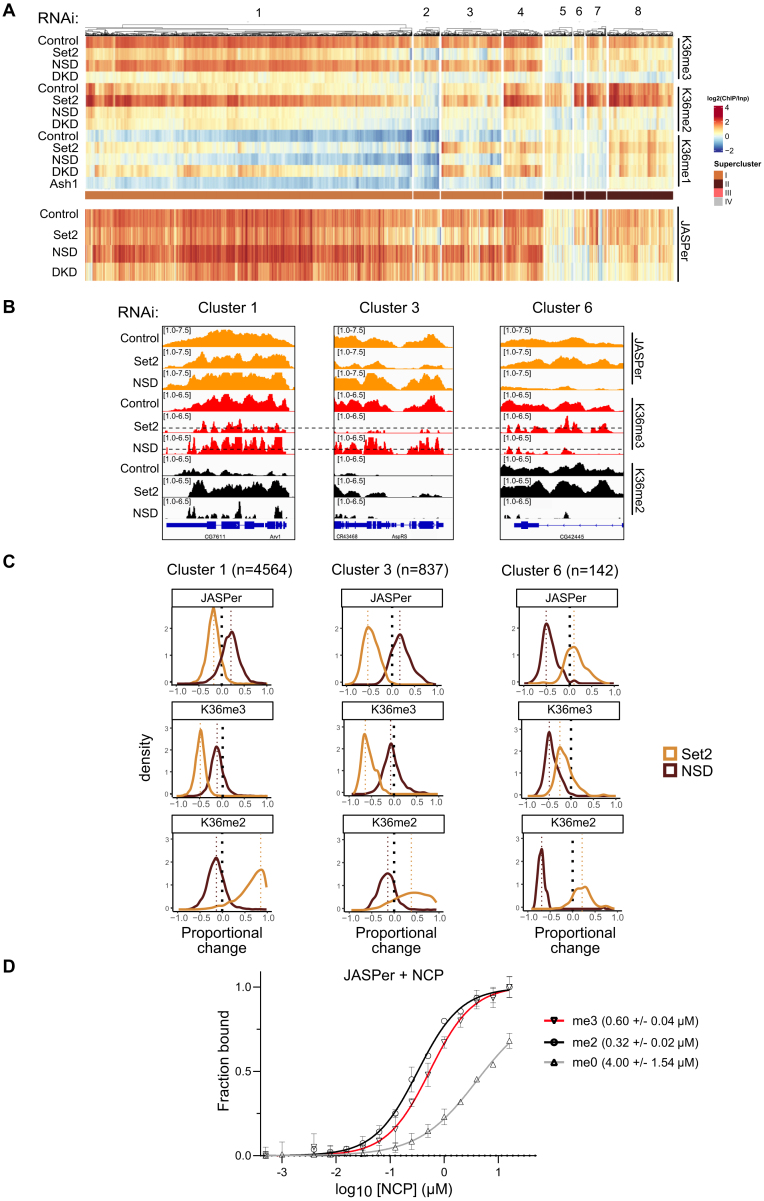
Robust binding of K36me3 readers above threshold K36me3 density. (**A**) Heatmap of gene body average ChIP signal for JASPer in indicated RNAi condition ordered according to heatmap from Fig. 4A. Note that supercluster III was excluded as it has very few genes bound by JASPer. (**B**) Genome browser profiles for three distinct genomic loci highlighting different responses of JASPer to Set2 RNAi and NSD RNAi within indicated clusters. RNAi condition and immunoprecipitation target are indicated to the left and right, respectively. The dotted line in K36me3 ChIP in Set2/NSD RNAi represents an arbitrary threshold below which strong reader binding reduction is observed, based on empirical observation of genome browser tracks. (**C**) Proportional change density plots representing direction and magnitude of change in gene body average ChIP signal for K36me2/3 along with reader JASPer for indicated representative clusters. Dashed line indicates the median value of each curve. (**D**) Equilibrium binding between recombinant JASPer and unmodified, dimethylated, or trimethylated nucleosomes, respectively, determined using MST. Error bars represent the standard deviation from the mean values obtained from *n* = 2 experiments. Calculated dissociation constants are indicated within parentheses.

Changes in K36 methylation patterns upon HMT depletion provide a unique opportunity to observe and compare the dependence of reader proteins on these marks. We explored the redistribution of exemplary reader proteins JASPer [36] (Fig. 6A) and MSL3 (ChrX-specific) [22, 35] (Supplementary Fig. S13E and F) upon HMT depletion for supercluster I and II genes, which comprise the majority of all reader binding events. The bulk levels of JASPer and MSL3 (inferred from other MSL complex proteins) were not affected upon Set2 or NSD depletion (Supplementary Fig. S13A).

Comparing the heatmaps of JASPer binding upon HMT depletion (Fig. 6A) revealed that JASPer reduction follows K36me3 in general, but not to the same extent. SC-I genes, where JASPer remains bound after significant loss of K36me3, are characterized by a strong gain of K36me2 upon Set2 depletion. For SC-II genes, JASPer binding is lost when both K36me2 and K36me3 are lost upon NSD depletion. Genomic profiles of representative genes in clusters 1, 3, and 6 illustrate the different scenarios inferred from the heatmap (Fig. 6B). These observations led us to hypothesize that JASPer may be attracted to both K36me3 and K36me2.

To explore this possibility further, we calculated proportional changes and compared the magnitude and direction of changes in reader binding and K36me2/3 signals upon HMT depletion at genes of clusters 1, 3, and 6, which show distinct dynamics as a population. At cluster 1 genes, where Set2 depletion leads to a strong reduction in K36me3 (median decrease of ∼50%) accompanied by a strong gain of K36me2 (median increase of ∼100%), JASPer binding is only mildly decreased (median decrease of ∼20%) (Fig. 6C, left panels). In contrast, at cluster 3 genes, where strong losses of K36me3 (median decrease of ∼70%) coincide with only a mild gain of K36me2 (median increase of ∼50%), JASPer binding is more compromised (median decrease of ∼55%) (Fig. 6C, middle panels), suggesting a lower degree of compensation by K36me2. In a third scenario highlighted by cluster 6 genes, both K36me2/3 are eliminated upon NSD knockdown resulting in proportional reduction of JASPer binding (median decrease of ∼60% for K36me3 and JASPer) (Fig. 6C, right panels). Interestingly, at these genes, despite the partial K36me3 reduction observed upon Set2 RNAi, JASPer binding is rather increased, possibly owing to increased K36me2. These findings support the hypothesis that JASPer binds both K36me2 and K36me3.

To quantitatively examine the contributions of K36me3 and K36me2 to JASPer binding at a gene level, we visualized the change in JASPer binding as a function of the change in K36me3 ChIP signal upon Set2 depletion (Supplementary Fig. S13B) for SC-I genes. Genes showing equiproportional reduction of JASPer in response to K36me3 reduction should lie close to the diagonal, while genes where JASPer binding is compensated by binding to K36me2 should be offset toward the left. The offset is observed for most SC-I genes and, further, genes that strongly lose JASPer binding are characterized by a very mild gain (or even a reduction) of K36me2 as denoted by the color of the dots. These observations suggest that at many genes JASPer binding relies significantly on K36me2. As predicted, at SC-II genes where K36me2 and K36me3 are both lost upon NSD RNAi, JASPer binding is lost proportionally (Supplementary Fig. S13C).

In light of the recent biochemical observation that the PWWP domain of LEDGF, the human ortholog of JASPer, can bind K36me3 and K36me2 with similar affinities [31], we measured the affinity of recombinant JASPer to K36me0/2/3 mononucleosomes by MST (Fig. 6D). JASPer was found to have a slight preference for K36me2 (*K*_D_= 0.32 ± 0.02 μM) over K36me3 mononucleosomes (*K*_D_= 0.60 ± 0.04 μM), whereas the affinity for unmodified nucleosomes was much lower (*K*_D_= 4.00 ± 1.54 μM), confirming that K36me2 can compensate for K36me3.

We explored whether the binding principles inferred from JASPer profiles also apply to the chromodomain protein MSL3. Because MSL3 binding is restricted to genes on the X chromosome, it cannot be displayed along with the reference heatmap that clusters all genes. Nevertheless, representative X-chromosomal genes of the reference clusters 1, 3, and 6 show that MSL3 binding correlates well with the combined presence of K36me3/me2 (Supplementary Fig. S13D and E). The corresponding density plots show that MSL3 binding follows the general pattern of JASPer (Supplementary Fig. S13F). At cluster 1 genes, MSL3 binding is virtually unperturbed, whereas at clusters 3 and 6 MSL3 binding is reduced along with K36 methylation in Set2 and NSD knockdowns, respectively. Because MSL3 binding profiles mirror that of JASPer, we hypothesize that MSL3 also binds to both K36me3 and K36me2. In summary, we suggest that MSL3 and JASPer dissociate from chromatin when the combined densities of K36me2 and K36me3 drop below a critical threshold.

The residual H3K36me2/3 at specific loci after HMT depletion presumably depends much on genomic context, and may differ in terms of transcription-associated nucleosome turnover and methylation/demethylation dynamics. Indeed, we observed that even after extended RNAi depletion of Set2 (Supplementary Fig. S14A), some genes in clusters 1 and 4 still maintain relatively high levels of K36me2/3 and JASPer recruitment. In line with these considerations, we found more elongating RNA polymerase II (ePol) at cluster 2/3 genes (“sensitive genes”) compared to “resistant” genes in clusters 1/4 (Supplementary Fig. S14B). Furthermore, we found that the putative K36 demethylase KDM2 [75], but not KDM4A, is enriched at “sensitive” clusters 2/3 genes. These findings suggest that robustness in K36me reader binding is determined by a complex interplay between dedicated HMTs and counteracting turnover mechanisms.

## Discussion

A variety of epigenetic processes have been linked to the methylation of histone H3 at lysine 36 in different organisms, mostly considering K36me2/3. Many of those processes influence the transcriptional output [5]. Although K36 methylation is generally considered as an active mark, it is relevant for silencing by DNA methylation [13, 14] and for silencing at facultative heterochromatin by H3K27me3 [15, 16]. In addition, K36me3 has been linked to genome stability (for review, see [76]) and HMTs establishing H3K36 methylation as well as K36me readers have been linked to cancer [77–79].

In mammals, HMTs that methylate K36 and corresponding reader proteins are numerous, suggesting partially redundant functions. To more easily uncover fundamental principles, we use the *Drosophila* model to explore the role of the three relevant HMTs Set2, NSD, and Ash1 in the genome-wide distribution of the three K36 methylation states. We also determine the genomic locations of two dedicated reader proteins: the chromodomain MSL3, a subunit of the male-specific DCC [35], and the PWWP protein JASPer, which tethers JIL1 kinase to active chromatin, where it reinforces the active state by H3S10 phosphorylation [36]. Importantly and in contrast to other methods, our optimized ChIP protocol enables improved mapping of K36 methylation and readers within constitutive heterochromatin (Supplementary Fig. S1A), which are often poorly solubilized in traditional ChIP protocols [80, 81], highlighting that they are not strictly active chromatin marks.

The mapping of each individual K36 methylation state in unperturbed conditions revealed complex distribution patterns. Yet, the full complexity of the profiles only became apparent when the changes upon single or combined HMT depletion were scored in a gene-centric manner and the resulting heatmaps subjected to unsupervised clustering. We hypothesize that the 11 clusters that emerged correspond to gene classes with shared properties and chromatin context. The grouping into superclusters served to outline broad features without getting lost in details. The heterogeneity of the loss-of-function phenotypes revealed local, context-dependent activities of the HMTs. Interestingly, a recent preprint adopting a very similar approach involving single and combinatorial knockouts of murine H3K36 methyltransferases echoes our observations regarding complexity in establishment of the K36 methylation landscape [82].

### Ash1 establishes H3K36me1 domains overlapping enhancers

Monomethylation is the most abundant methylation state of K36 in *Drosophila* [71], but not well studied in other organisms. We found that K36me1 represents a defined chromatin state in regions with enhancer features, often in introns of differentially expressed genes. These sites cover ∼15% of the genome and may correspond to the most densely K36-methylated domains, according to the relative abundance of the three methylation states [71]. Remarkably, those sites bear little K36me2/me3 (see exception below) and K36me1 appears often rather complementary to me2/me3 signals. Evidently, K36me1 is not only a methylation intermediate toward K36me2/3, but may have its own function at enhancers, which needs to be further explored. To our knowledge, readers that selectively recognize K36me1 are not known.

Most of K36me1 is placed by Ash1, an HMT that so far is mostly described as a K36 dimethylase important for restricting Polycomb repression in flies and mammals [15, 83]. At other sites, such as a fraction of supercluster III genes, NSD and Set2 may convert Ash1-dependent K36me1 into K36me2/3. The function of this locus- and context-specific methylation pathway at a large fraction of genic K36me1 enhancer domains remains to be explored.

### H3K36 methylation by NSD and Set2 represents distinct pathways

Contrary to our expectations, in steady-state conditions in S2 cells, NSD and Set2 act largely independently of each other at distinct chromatin domains and different genes. Set2 catalyzes K36me3 predominantly within intron-poor, highly transcribed housekeeping genes, while NSD catalyzes K36me2/3 in a heterochromatic environment as well as at numerous euchromatic, weakly transcribed genes with many introns and cell-specific expression profiles. Our data suggest that, in general, both NSD and Set2 can methylate nucleosomes *de novo* without prior “pioneering” methylation by another enzyme. In addition, Set2 may also be able to add a methyl group to K36me2 (generated by NSD) in certain contexts, as also observed *in vitro* [37]. Furthermore, we find that NSD is not only a K36 dimethylase but also capable of generating K36me3 especially at heterochromatin in *Drosophila*, which resonates with a recent report in mice [84]. These results are in line with recent studies documenting distinct loss-of-function phenotypes of Set2 and NSD in flies [25, 28]. Moreover, these notions are corroborated by our reanalysis of independent mouse datasets, where we find that the mammalian K36 HMTs contribute to separate methylation profiles at distinct gene classes.

### Targeting, regulation, and function of H3K36 methyltransferases

Unsupervised clustering of the K36 methylation changes at genes upon HMT depletion revealed a clear functional partitioning of the three HMT activities, which is, however, not exclusive. Each HMT appears to be most strongly enriched at loci, where depletion studies reveal their activity (Set2 in supercluster I genes, NSD at supercluster II, and Ash1 at supercluster III). However, they can also be detected at other sites, where their contribution is more restricted. For example, NSD is detected at supercluster I genes where K36 methylation largely depends on Set2. Conversely, Set2 can be mapped to supercluster II genes, where most K36me depends on NSD. These observations suggest that the functions of K36 HMTs can be regulated at two different steps, their targeting and the activation of enzymatic activity. This is reminiscent of yeast Set2, which is targeted to transcribed genes by interaction of the conserved SRI domain with the elongating RNA polymerase II. The SRI domain directly contributes to activation since its deletion abolishes K36me3 and reduces H3K36me activity *in vitro*. Further facilitation of Set2 methylation activity is achieved in the presence of monoubiquitinated Lys123 of H2B on nucleosome substrates (for review, see [85]).

While Set2 is recruited to transcribed chromatin by interaction with the transcription machinery, it is less clear how NSD is targeted. It has been reported to be recruited to Beaf-32-bound active promoters [43], but we found no systematic association between Beaf-32 peaks and promoters of SC-II NSD-dependent genes (not shown). The molecular basis of NSD localization to heterochromatin was hypothesized to depend on HP1 [38]; however, preliminary experiments (unpublished observations, and personal communication from D. Atkinson and T. Jenuwein) as well as another recent study [86] argue for an HP1-independent targeting mechanism. Sun *et al.* described a catalytic activity-independent role for the mouse NSD1 in promoting enhancer function by localizing to active enhancers [68]. In contrast to these observations, we do not observe NSD at H3K27ac/H3K4me1 domains. For mammalian NSD enzymes, an autoinhibitory state is relieved by binding to nucleosomes [87] to trigger activation, while NSD2 has been shown to be inhibited by H1 in nucleosomes [88]. However, one conserved feature of all NSD orthologs is the presence of two PWWP domains, which could potentially modulate the activity of NSD and be implicated in a maintenance function. K36me3 is a relatively rare mark [71]; thus, it is also conceivable that NSD acts as a “maintenance” methyltransferase by associating with a K36me2/3 mark. Such a scenario may serve to increase the local density of K36me3, to place K36me2 in the vicinity of K36me3 “seeds” or to preserve the signature of active chromatin at poorly transcribed genes. Altogether, the targeting, activation, and molecular function of *Drosophila* NSD, potentially through different mechanisms at euchromatin and heterochromatin, remain to be addressed in future studies. The function reported for mammalian NSD to establish faithful DNA methylation [13, 89] will not be conserved in *Drosophila* that lacks DNA methylation-related machinery. The determinants of genomic distribution of Ash1 have not been elucidated, but it has been shown that *Drosophila* Ash1 is activated by MRG15 [41, 44].

While our study thoroughly documents the changes in K36 methylation occurring upon depletion of K36 HMTs, the consequences of altered K36 methylation are less clear. We investigated two phenomena commonly discussed in the context of K36 methylation, namely transcriptional regulation and counteracting the spreading of Polycomb-dependent K27me3. In agreement with previous studies [26], we found that changes of K36 modification and transcription upon HMT depletion were poorly correlated, precluding a causal link between K36 methylation and transcription. We are aware of potential indirect effects due to HMT-dependent methylation of non-histone proteins, such as methylation of alpha-tubulin by SETD2 [90]. Any effect of K36 methylation on transcription is likely to be mediated by the nature (and combination) of functionally diverse K36me readers bound at a particular gene locus and may involve RNA processing [26]. Further, our preliminary observations also suggest that K36 HMTs, especially NSD and Ash1, independently counteract K27me3 spreading at some developmental genes. However, this effect is restricted to a fraction of all genes carrying K36 methylation, suggesting that counteracting K27me3 is not the sole function of K36 methylation.

### Indirect communication between HMTs

In at least two instances, we observed that the depletion of one HMT led to increased methylation activity of remaining HMT(s). Often, this effect is subtle and sensitive to the normalization approach, making it hard to distinguish technically. However, the strong increase of NSD-dependent K36me2 at SC-I genes upon Set2 depletion has been consistently observed in our dataset, and similar effects can be observed across two different mouse datasets [13, 68]. Such an effect may be explained by local competition for a shared substrate, such as SAM. Indirect communication between HMTs of this kind may lead to underestimation of co-dependences between them, in particular at genes where they colocalize, if one activity is increased when the other one is depleted. However, it also highlights one possible mechanism that ensures robustness of H3K36me2/3 distribution upon metabolic perturbation. Interestingly, recent studies in mammals have linked H3K36me3 to the methionine cycle and vitamin B_12_ levels, which determine SAM availability, in reprogramming and cell differentiation [91, 92].

### Robust readout of H3K36 methylation by readers bearing chromo and PWWP domains

At transcribed chromatin, the function of K36 methylation is mediated by dedicated reader proteins, of which we considered two, MSL3 and JASPer, which associate with methylated K36 with different domains. Both proteins behave similarly in our experiments. For MSL3, our systematic analysis extends previous studies [35, 42], which either did not involve high-throughput sequencing or utilized Set2-deficient fly larvae. Our RNAi approach provides a spectrum of intermediate K36me2/3 levels, which we exploit to obtain quantitative insights into reader binding.

We show that JASPer binds nucleosomes with K36me2 and K36me3 with similar affinity, as described for its mammalian ortholog LEDGF [31]. This may explain why JASPer remains associated with genes that significantly lose K36me3 but accumulate K36me2. The ability of readers to recognize both states may ensure that transcribed chromatin is reliably bound upon changes. Furthermore, the fact that K36me3 is enriched at exons, while K36me2 is more prevalent at introns may assure that all aspects of transcribed chromatin are equally well addressed.

It appears that the chromatin interactions of both, the chromodomain reader MSL3 and the PWWP reader JASPer, depend on critical densities of combined K36me3/K36me2, regardless of which enzyme catalyzes the methylation. We consider that the combined capacity of the K36me3/2 marks normally exceeds the requirements for productive tethering of the reader proteins, conferring robustness in reader binding against fluctuations during local turnover of histone or methyl mark. While we formally estimated the contribution of K36me2, we cannot exclude the contribution of other chromatin features to reader binding in our model system. In line with this, despite very strong reduction of both K36me2/3 in double knockdowns, JASPer binding is retained to some degree at many genes. Loci where reader binding appears more sensitive to Set2 or NSD depletion may have a higher turnover of H3K36me2/3 and/or of the nucleosomes themselves. Corresponding physiological conditions may include changes in metabolic conditions (methionine or vitamin B_12_ availability; see earlier) especially for Set2-dependent genes and expression changes especially for NSD-dependent differentially expressed genes.

### Methylation readout at heterochromatin

We found that K36me3 partitions between eu- and heterochromatin. The presence of K36me3, a mark globally associated with active chromatin, at H3K9-methylated heterochromatin has also been observed in other studies [3, 84] and described in human datasets [93, 94]. Such overlap of active with more repressive chromatin features may not simply be due to population heterogeneity since engineered dual readers validated the existence of such dual domains [84, 95] and have been proposed to bookmark poised enhancers genome-wide in mouse [84]. In *Drosophila*, the co-occurrence of those marks is mostly observed at genes embedded at PCH, which also recruit JASPer and MSL3 readers. However, the relationship between K36me3/2 at PCH and reader binding is complicated as we also observe many instances of heterochromatic K36me2 domains that fail to recruit JASPer. Another heterochromatin modification/component may hinder binding to K36me2 locally. We thus speculate that the selective recruitment to K36me2/3 *in vivo* may be modulated in the chromatin context.

### Limitations of our study

Our systematic and comprehensive approach necessitated to focus on a simple cellular model. The fundamental principles we uncovered are likely to be modulated in cell-specific ways in the fly organism, where the expression of HMTs and KDMs varies spatiotemporally. For instance, NSD is maternally deposited and present during early development, while Set2 contribution starts at NC5 with the minor zygotic gene activation.

The RNAi approach results in strong reduction of target protein levels, but does not eliminate it entirely. For non-processive methyltransferases, the efficiency with which the three methyl groups are added to the same lysine may get progressively lower. Upon RNAi, there may still be enough enzyme to add two methyl groups but not enough to bring it to me3. In ChIP-seq, this may look like the knockdown leads to the “substitution” of H3K36me3 by H3K36me2. This may be an alternative explanation to the observed appearance of K36me2 within supercluster I genes, which we interpreted as differences in turnover of K36me3.

Due to a lack of ChIP-grade antibody, we did not directly map Set2, but infer the genomic Set2 localization from the profiles of elongating RNA pol II.

Our antibody-based approach is blind to the H3 variant that bears the K36me mark and it is likely that the replication-independent histone H3.3 variant contributes to the observed ChIP signals. H3.3 is enriched at active chromatin, transposons, and the hyperactive male X chromosome in *Drosophila* [96]. Since H3.3 accounts for ∼20% of all H3 and is placed where nucleosomes turn over, it is possible that a large fraction of the K36me marks we score reside on the variant [97].

The genomic annotation of introns and exons is based on whole-fly RNA datasets. It is possible that the long introns methylated by NSD might contain unannotated tissue-specific exons. Alternatively, K36me2/3 at these long introns may mark cell-specific regulatory domains (e.g. enhancers).

## Supplementary Material

gkaf202_Supplemental_File

## Data Availability

The raw sequencing files in fastq format and the summarized genome browser tracks in bigwig format are available in the GEO database (GSE253391). Reference tracks can be viewed on UCSC Genome Browser using the following link: https://genome.ucsc.edu/s/Muhunden/Jayakrishnan_2025. Custom code for analysis of data is available on Zenodo (https://doi.org/10.5281/zenodo.14673023). Western blots and immunofluorescence images used for quantifications in Fig. 2 and Supplementary Fig. S2 are available on Zenodo (https://doi.org/10.5281/zenodo.10514405).

## Supplementary data

Supplementary data is available at NAR online.
