# Supplementary material for: Genomic context-dependent histone H3K36 methylation by three Drosophila methyltransferases and implications for dedicated chromatin readers
Source: Nucleic Acids Res. 2025 Apr 1;53(6):gkaf202. doi: 10.1093/nar/gkaf202 (PMC12262051; doi:10.1093/nar/gkaf202)
Supplement: gkaf202_Supplemental_File [file gkaf202_Supplemental_File.pdf]

Supplementary Figure S1

A

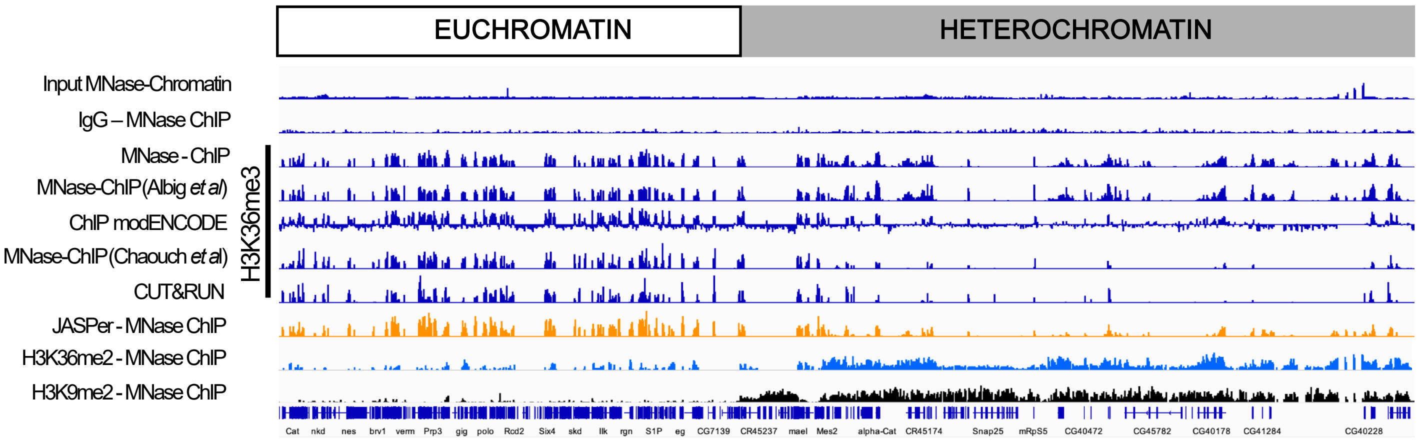

B

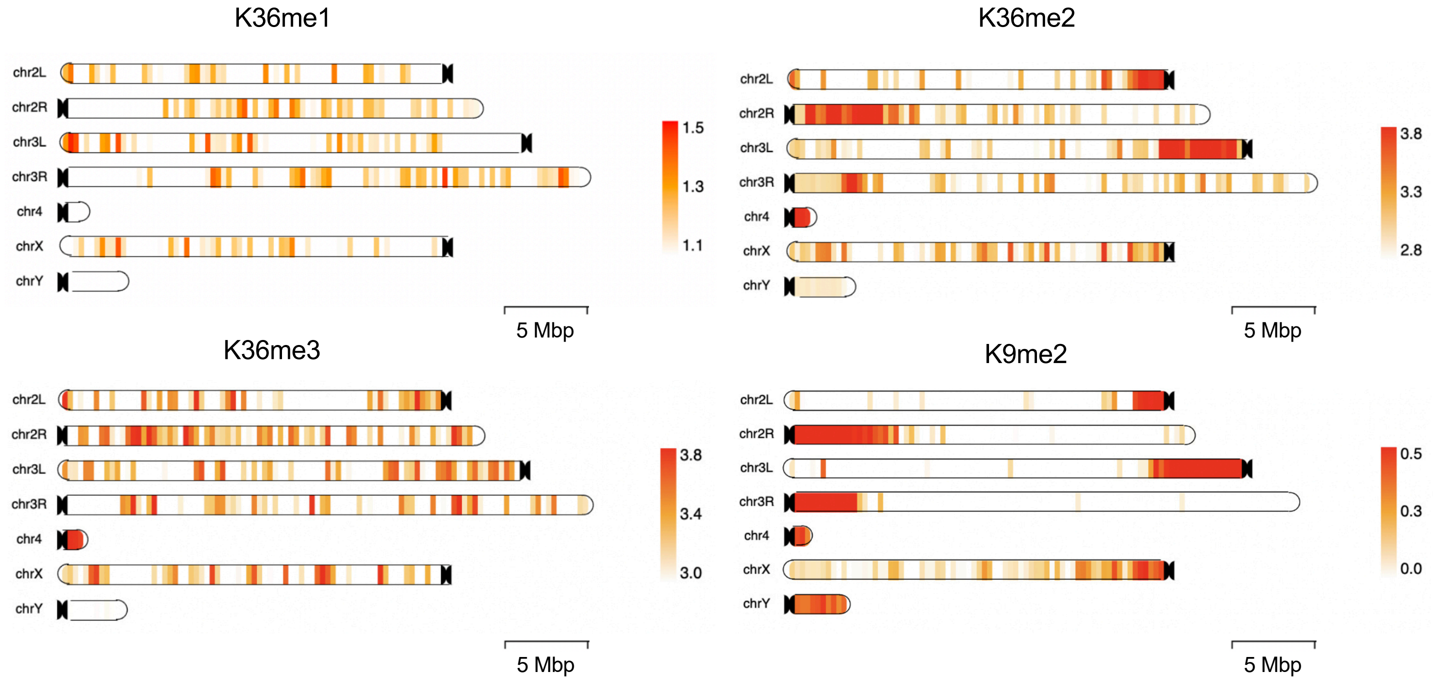

C

	Fraction genome coverage (%)	Bonnet *et al* abundance Late embryo (copies/1C genome)	Density of mark (per n nucleo-somes)	Feller *et al* abundance S2 cells (% of H3K36)
me1	12.4	124,000	9 in 10	2
me2	23.3	64,000	1 in 4	1.1
me3	18.1	6,000	1 in 28	0.3

D

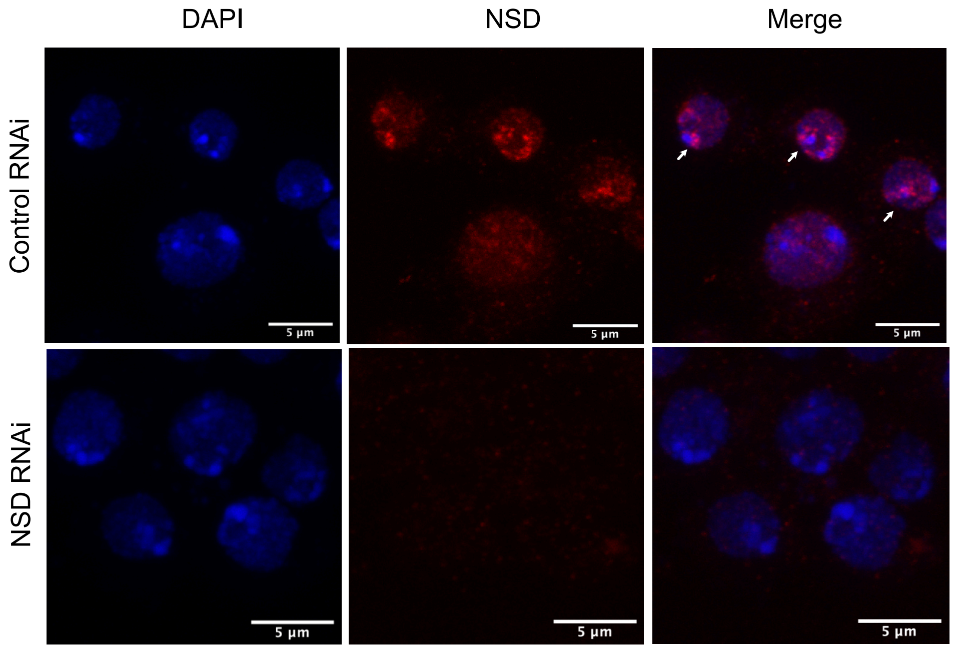

Supplementary Figure S1

Supplementary Figure S1 : K36 modifications and HMTs are differentially distributed in male *Drosophila* cells.

A) Genome browser profiles highlighting euchromatin bias introduced by the K36me3 mapping method. Comparison of different ChIP methods: MNase-ChIP: this work and Albig et al., (36) (includes mild shearing); CUT&RUN: this work; MNase-ChIP Chaouch et al., (50): native MNase-ChIP without crosslinking or shearing; ChIP modENCODE: Bioruptor shearing. IgG ChIP as well as chromatin input serve as negative controls. Colocalization of K36me3 signal with reader JASPer (MNase-ChIP) confirms specific signal. K36me2 coverage is provided for reference. H3K9me2 ChIP coverage demarcates heterochromatin and euchromatin.

B) ChromoMaps representing steady-state enrichment of K36me1/2/3 in 10-kbp genomic bins for all *Drosophila* chromosomes. The H3K9me2 chromoMap serves as a reference for mappable pericentric heterochromatin domains. Note that continuous ChIP coverage is represented instead of peaks (representing regions of strongest signal) used in Figure 1B.

C) Genomic coverage of K36me1/2/3 peaks and estimated densities based on Bonnet et al., (71). It should be noted that the density of K36me2 at heterochromatin is likely much higher than euchromatin.

D) Immunofluorescence confocal microscopy of S2 cells treated with dsRNA against either GST (control) or NSD RNAi. Staining for DNA, NSD and merged images are shown. Arrowheads indicate chromocenter-proximal speckles of NSD. Scale bar is 5 µm.

Supplementary Figure S2

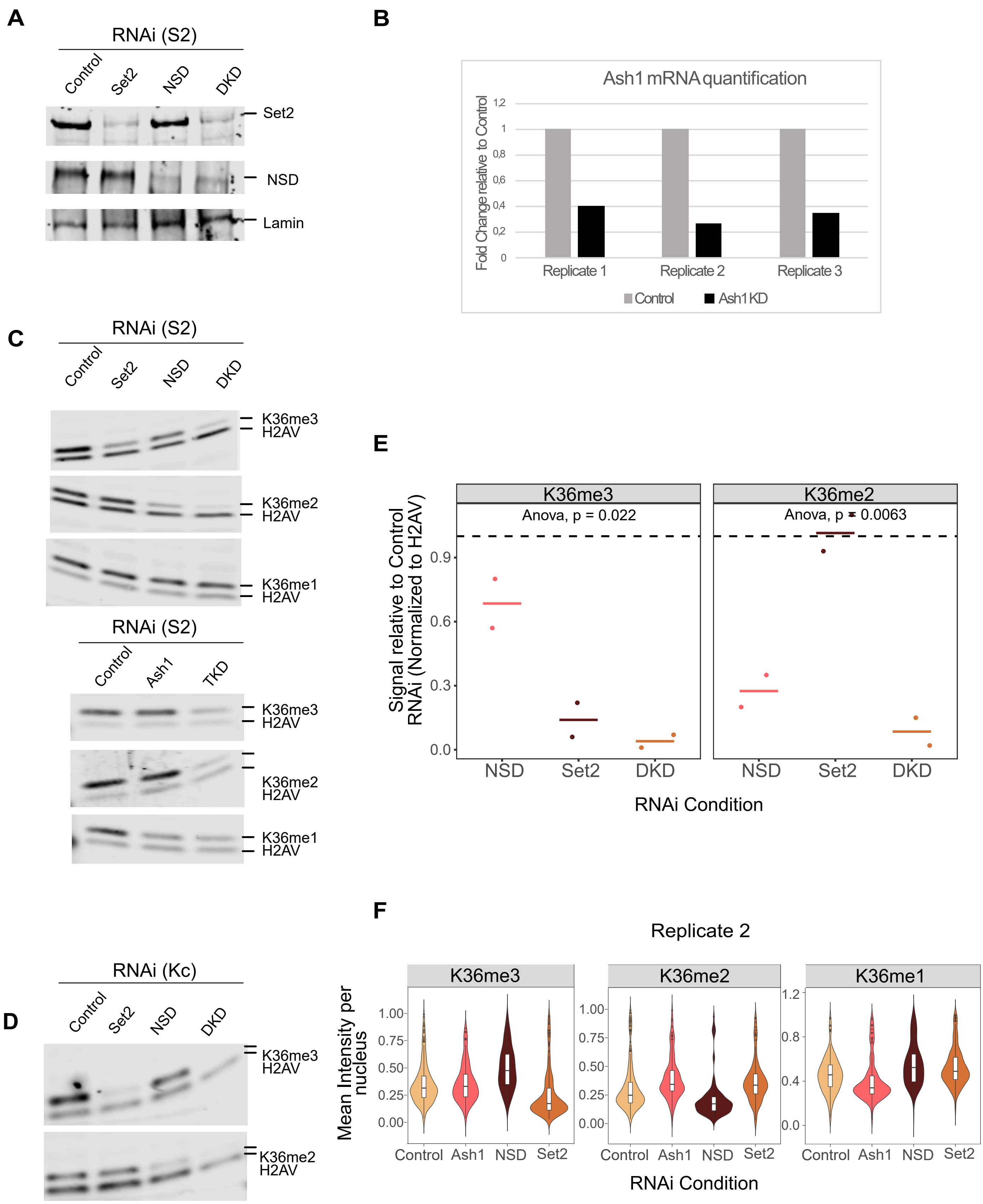

Supplementary Figure S2

Supplementary Figure S2 : HMT depletions lead to similar alteration of bulk K36 methylation in male and female cells.

A) Representative Western blot documenting the depletion of indicated HMTs in whole cell extracts from S2 cells treated with corresponding dsRNA. α-Lamin served as loading control. DKD: double knock down of Set2+NSD.

B) RT-qPCR quantification of mRNA extracted from S2 cells treated with Ash1 dsRNA (N=3).

C) Representative Western blots of K36 methylation marks used for quantification in Figure 2A.

D) Representative Western blots of K36 methylation marks from Kc cells treated with GST (Control), NSD, Set2 or NSD+Set2 (DKD) dsRNA.

E) Quantification of Western Blot analysis from Supplementary Figure S2D (N=2).

F) Quantification of immunofluorescence images (n=~500 nuclei) from 2nd biological replicate. First replicate presented in Figure 2C.

Supplementary Figure S3

A

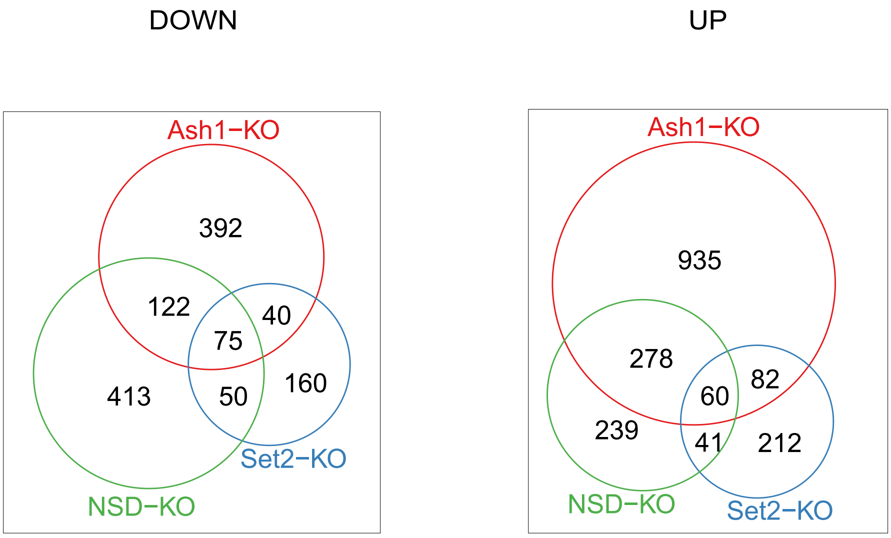

Supplementary Figure S3 : Poor overlap in transcriptomes of HMT Knockout *Drosophila* larval brains.

A) Venn diagram representing overlap between significantly up-/downregulated genes ($p<0.05$, log2FC < -0.32 or log2FC > 0.26 (i.e. increased or decreased by at least 20%) from HMT knock-out (KO) Fly Larval Brain RNAseq data (38).

Supplementary Figure S4

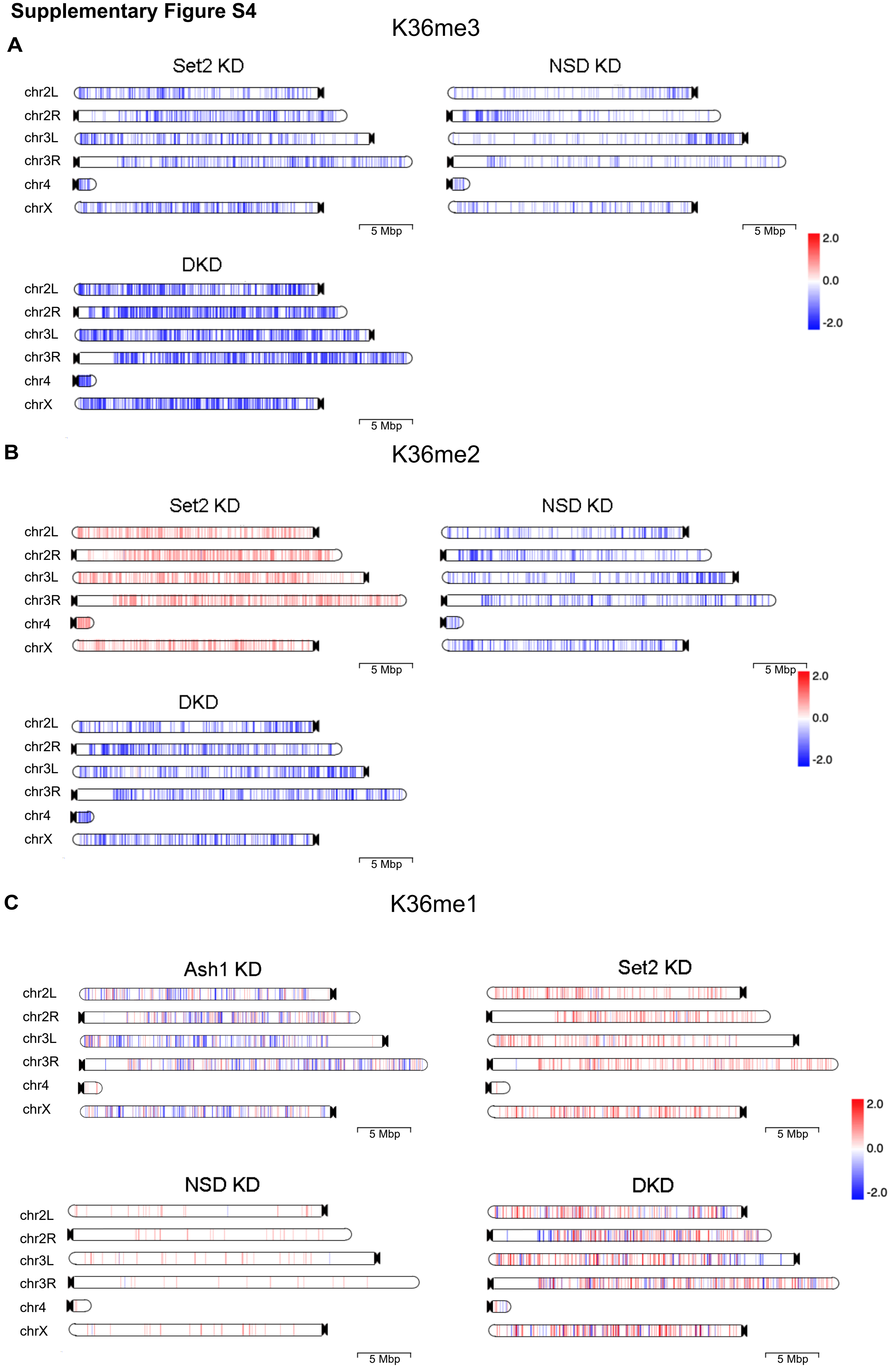

Supplementary Figure S4

Supplementary Figure S4 : HMT Knockdown affects K36me1/2/3 in predominantly distinct genomic compartments.

A-C) K36me3/2/1 chromoMaps representing regions of significant difference as derived from csaw analysis at 2-kbp resolution for all *Drosophila* chromosomes across all tested RNAi conditions. The color of the regions (as indicated by the common scale in Figure 3B) represent log2-transformed value of number of normalized reads in RNAi condition relative to control condition.

Supplementary Figure S5

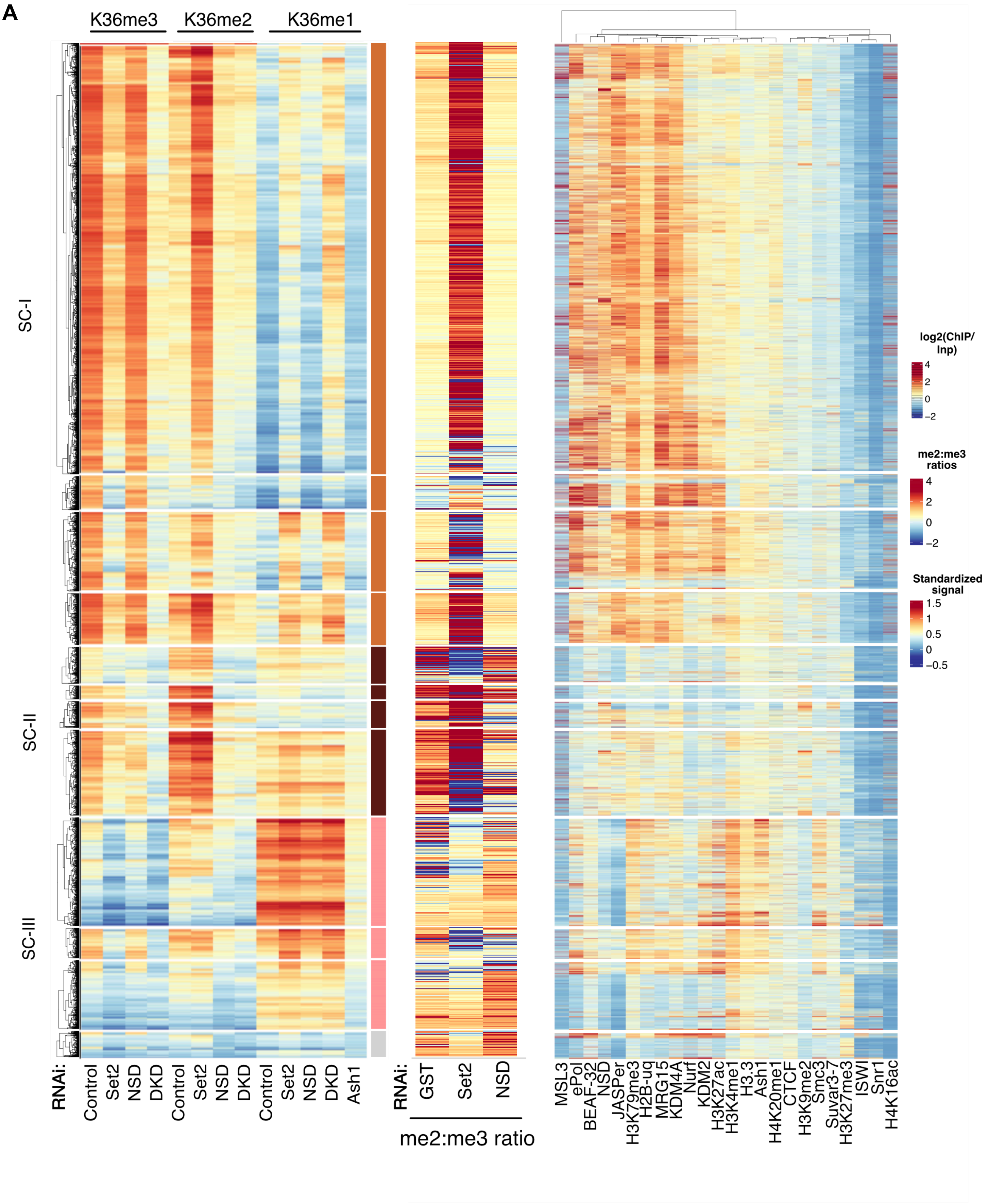

Supplementary Figure S5 : Clusters defined by K36 methylation patterns are correlated to distinct me2:me3 ratios as well as chromatin factor occupancy

A) Left: Main heatmap defined in Figure 4A representing gene clusters of specific K36 methylation patterns.
Middle: Heatmap representing K36me2:K36me3 ratios upon RNAi of indicated factor ordered according to main heatmap.
Right: Heatmap of standardized average gene body signal of chromatin factors represented in Figure 1C ordered according to main heatmap. Columns were automatically clustered based on similarity. All features were scaled to the uniform range (0,1) by min-max normalization where min and max refer to bottom and top 5% genomic ChIP signal respectively.

Supplementary Figure S6

A

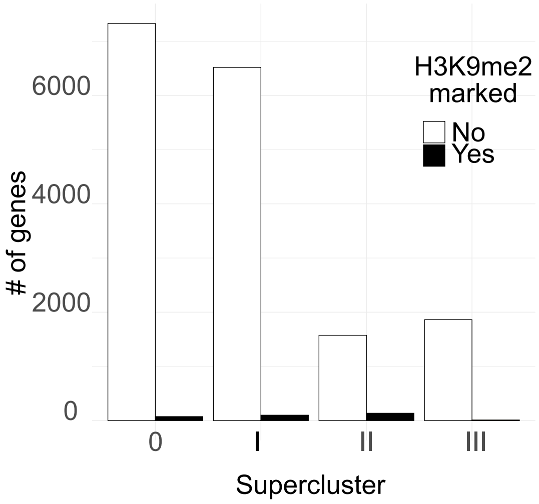

Supplementary Figure S6 : NSD-dependent methylation is present on many euchromatic genes.

A) Quantification of proportion of Eu-/Heterochromatic genes for each supercluster-specific genes based on overlap with K9me2 peaks.

Supplementary Figure S7

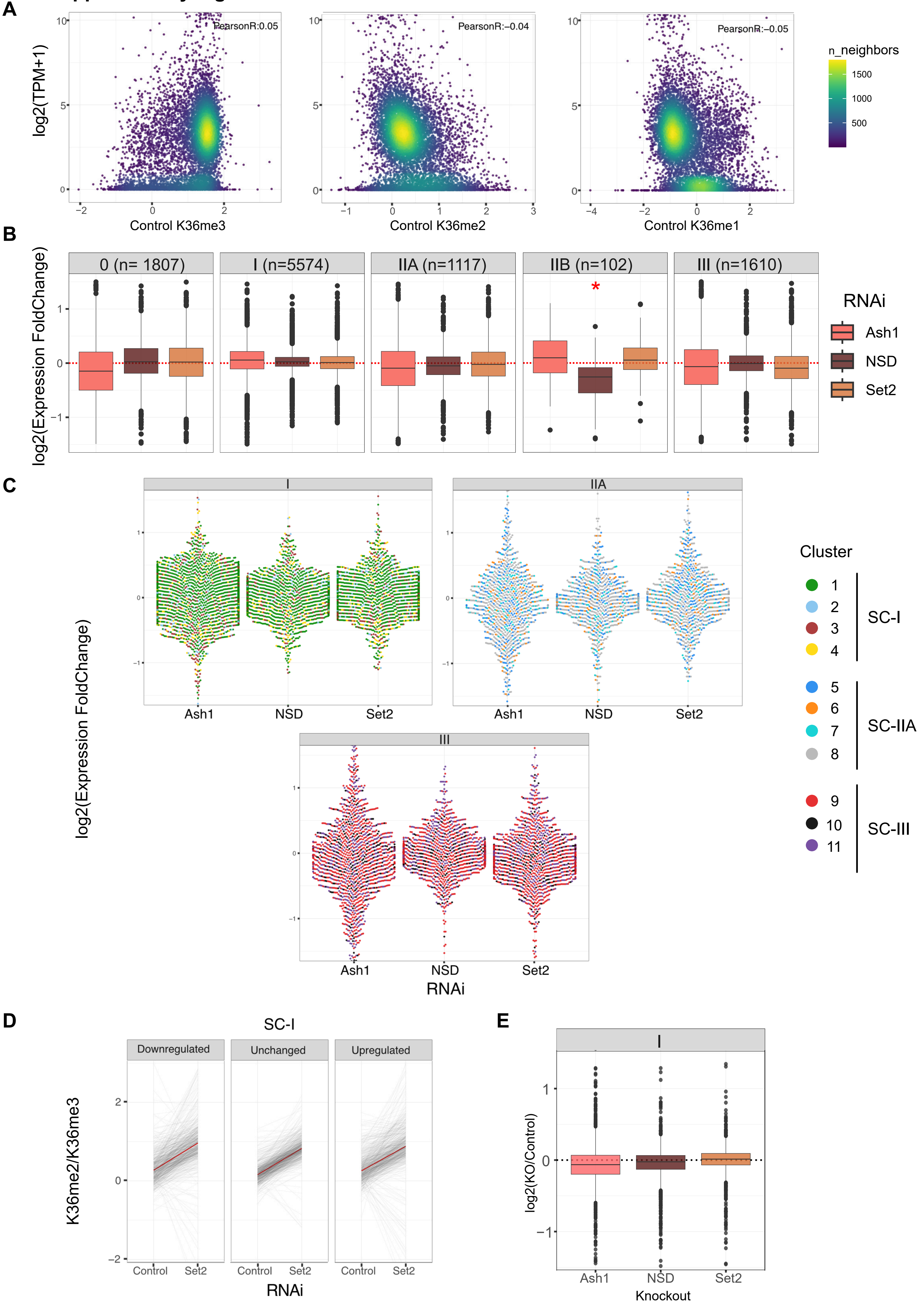

Supplementary Figure S7 : Transcriptional changes occurring upon HMT RNAi correlate poorly with changes in K36 methylation

A) Scatter plots showing lack of correlation (PearsonR value) between transcription (log2- transformed S2 RNAseq TPM values) and genic K36me3, K36me2 and K36me1 signal (as indicated). Overlayed color indicates density of points in the neighbourhood. Only genes containing atleast one K36me modification (same as heatmap Figure 4A) were used.

B) Boxplots representing RNA-seq transcriptional change (log2(RNAi/Control Expression)) upon RNAi of indicated factor for genes of superclusters I-III. Supercluster 0 consists of genes lacking any detectable K36 methylation. n = Number of genes within each class. Only one biological replicate per sample was available from corresponding source data. Note that the discrepancy in the number of supercluster genes compared to numbers represented in Figure 4B arises due to filtering of silent genes (zero RNA-seq reads in all biological samples) for differential gene expression analyses. Two-sided one sample t-tests were performed for each 'box' i.e. RNAi - supercluster combination (null hypothesis: group expression change = 0) and significance adjusted for multiple testing. ' * ' represents the only group that passed the threshold (pvalue<0.05, absolute(group mean log2-expression change)>0.15).

C) Beeswarm plots representing transcriptional change upon HMT RNAi for individual genes (dots) belonging to indicated superclusters. Color of dots indicate the gene cluster annotation (1-11) as also shown in Figure 4A.

D) Lineplots demonstrating change in K36me2:K36me3 ratios for individual genes upon Set2 RNAi for following gene groups-
(Left): Top 10 percent most downregulated (log2FC < -0.21) supercluster I genes upon Set2 RNAi (n=599)
(Middle): 10 percent most expression unchanged genes (.ie. log2FC ~ 0) (n=547)
(Right): Top 10 percent most upregulated (log2FC > 0.24) supercluster I genes upon Set2 RNAi (n=584)
Red line connects median ratios in Control and Set2 conditions

E) Boxplots representing RNA-seq transcriptional change (log2(KO/Control Expression)) upon knockout of indicated factor in fly brains for supercluster I genes. Note that supercluster I genes were further filtered to retain only genes that show highly correlated expression in both Fly brains and S2 cells (n= ~ 3400)

While previous studies have demonstrated that presence of K36me3 on gene bodies is correlated to active transcription, the prevailing concept is that K36 methylation is not directly involved in transcription regulation, but may indirectly affect transcription through recruitment of reader proteins and crosstalk with repressive histone modifications. We anticipate that transcriptional regulation by K36me will strongly depend on the nature (and combination) of K36me readers bound at the particular gene locus. In *Drosophila*, there are about a dozen readers which have very diverse roles in regulating transcription. For example, reader MSL3 positively regulates transcription by directing the MSL acetyltransferase complex to X chromosomal genes (Sural et al., 2008). On the other hand, reader Rpd3s tethers a repressive complex to suppress cryptic transcription (Carrozza et al., 2005). Given that the binding of all K36me readers is likely perturbed to some extent upon HMT knockdowns, along with unknown changes in other factors like histone variants (H3.3 vs H3.2), demethylases localization, methylation of non-histone proteins etc., we predict a highly heterogenous effect on transcription.

Firstly, to verify the assertion that K36me3 is correlated to active transcription, we plotted the relationship between mean genic K36me3/2/1 signal and RNA-seq TPM in unperturbed S2 cells (Supplementary Figure S7A). The high K36me3 signal at expressed genes did not scale with transcription levels (represented by the vertical cloud). K36me3 may thus be considered as a binary indicator of ON/OFF status of a given gene rather than a mark that promotes transcription.

We then attempted to correlate changes in gene expression to changes in K36me upon HMT KD in S2 cells. To explore this, we utilized published S2 cells RNA-seq datasets for Ash1 RNAi (Huang et al., 2017) and Set2, NSD RNAi (Depierre et al., 2023). We calculated a log2-Fold change in gene expression for each RNAi with respect to their respective controls and visualized these values as a boxplot for each supercluster defined in Fig 4 (Supplementary Figure S7B). To reiterate, these superclusters reflect groups of genes that show relatively uniform changes in K36me upon HMT RNAi. If K36me has a consistent effect on gene transcription, HMT RNAi should result in a unidirectional outcome (consistent up- or down regulation) on gene expression. Inspection of the boxplots reveal that while we do observe outliers, the majority of the genes (indicated by the interquartile box) in all gene clusters do not show a consistent upward or downward trend in response to HMT RNAi, suggesting that K36 methylation changes are poorly predictive of gene expression changes. One exception appears to be SC-IIB (NSD-dependent heterochromatic) genes, where around 80% of genes are consistently decreased in expression upon NSD RNAi. Consistent with this, a previous report documented that NSD positively regulates the expression of a subset of heterochromatic genes (Saha et al., 2020). Further, transcriptional defects occurring upon RNAi of a particular HMT (indicated by outlier points representing differentially expressed genes) are not restricted to the supercluster where the HMT predominantly performs methylation, sometimes even affecting genes lacking any K36me (SC-0), suggesting indirect effects.

Clusters within the larger supercluster often differ from one another based on Control as well as turnover intermediate K36me levels (for example, see K36me1 differences in clusters 2 and 3 in Figure 4A), which may affect transcriptional changes differently. Hence, cluster identity (rather than the supercluster) may correlate better with transcriptional changes. To inspect this, we visualized transcriptional change as beeswarm plots for selected superclusters, which are essentially violin plots representing each data point (gene) overlaid by the color of the clusters. We can observe that the colors of the points are rather homogeneously distributed throughout the entire beeswarm, confirming that cluster identity also correlates poorly to transcriptional change.

Another hypothesis is that the ratio of K36me2 to K36me3, rather than their absolute levels, may explain transcriptional changes as the ratio has been previously implicated in transcriptional elongation. These ratios are visualized in the heatmap in Supplementary Figure S5. To test this, we selected top 10% most up- and downregulated SC-I genes (along with similar number of unchanged genes as a control set) in Set2 RNAi condition and followed how genic me2:me3 ratio changes upon Set2 RNAi as line plots. We observe that the me2:me3 ratios strongly increase in all three gene sets (which differ in the nature of transcriptional change), arguing that these ratios are poorly predictive of transcriptional change as well. Analysis of NSD-dependent transcriptional changes correlated to NSD-dependent me2:me3 ratios also leads to the same conclusion (data not shown). Taken together, we conclude that neither absolute K36me2/3 amounts nor the me2:me3 ratios explain transcriptional changes occurring upon HMT depletion.

Our RNAi approach often results in incomplete removal of K36me3 and appearance of lower K36me states, which may act functionally redundantly with K36me3 (see Figure 6). Thus, the effect of RNAi on transcription may be dampened. To verify the robustness of these observations using an orthogonal dataset, we revisited the fly larval brain HMT knockout RNA-seq data from Lindehell *et al.*, and performed a similar analysis as Supplementary Figure S7B. These knockouts should result in near complete elimination of K36me deposited by the corresponding HMT. Since the transcriptomes of S2 cells and fly brains differ substantially, we restricted our analyses to housekeeping genes which show highly correlated expression in both systems which are predominantly enriched in supercluster I. Since housekeeping genes have generally conserved regulatory mechanisms across different tissues (by the definition of ‘housekeeping’), it can be assumed with reasonable confidence that these genes are methylated at H3K36 by Set2 in the fly larval brain as well. We can now assess the effect of Set2 knockout on gene expression for these ~3500 genes (Supplementary Figure S7E) which confirms that K36me loss has no major effect on transcription. Thus, Set2 KO (or KO of any other HMTs) doesn’t have a unidirectional effect on gene expression which cannot be easily explained by overall reduction in K36me3 at these genes.

Supplementary Figure S8

A

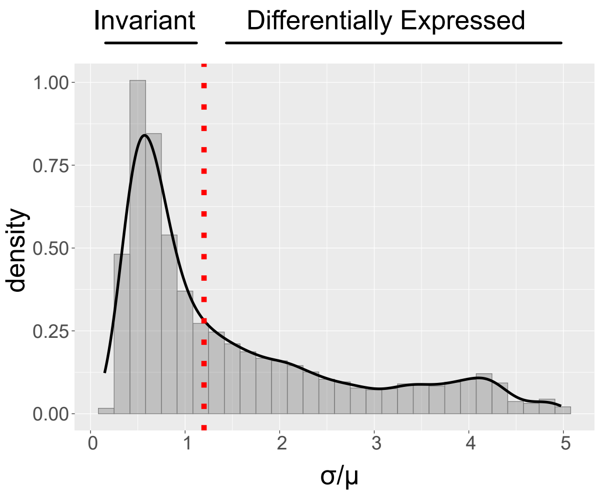

Supplementary Figure S8 : Classification of housekeeping (tissue invariant) and developmental (differentially expressed) genes based on FlyAtlas.

A) Distribution of σ/μ values (σ = Expression Standard Deviation, μ = Expression Mean; calculated across 25 FlyAtlas Tissues). The red line represents the arbitrary threshold distinguishing tissue invariant and differentially expressed genes. Of note, genes with very low expression in all 25 tissues were automatically classified as differentially expressed as they are likely expressed in only a highly specialized cell type.

Supplementary Figure S9

A

N=1

H3K27me3

Bin Classification (change upon RNAi)	n
Unchanged	14629
Ash1 lost	29
Ash1 gained	149
NSD lost	37
NSD gained	402
Set2 lost	17
Set2 gained	42

H3K9me2

Bin Classification (change upon RNAi)	n
Unchanged	2754
NSD lost	19
NSD gained	15

B

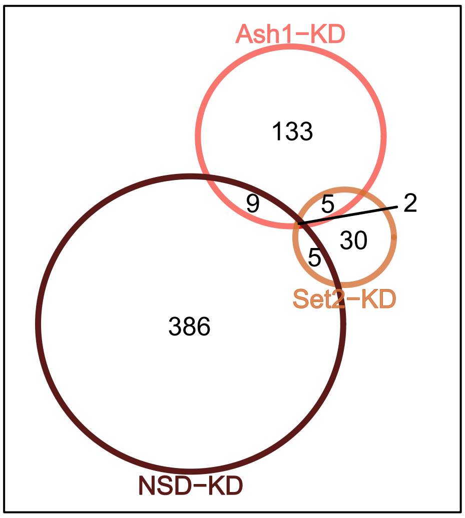

C

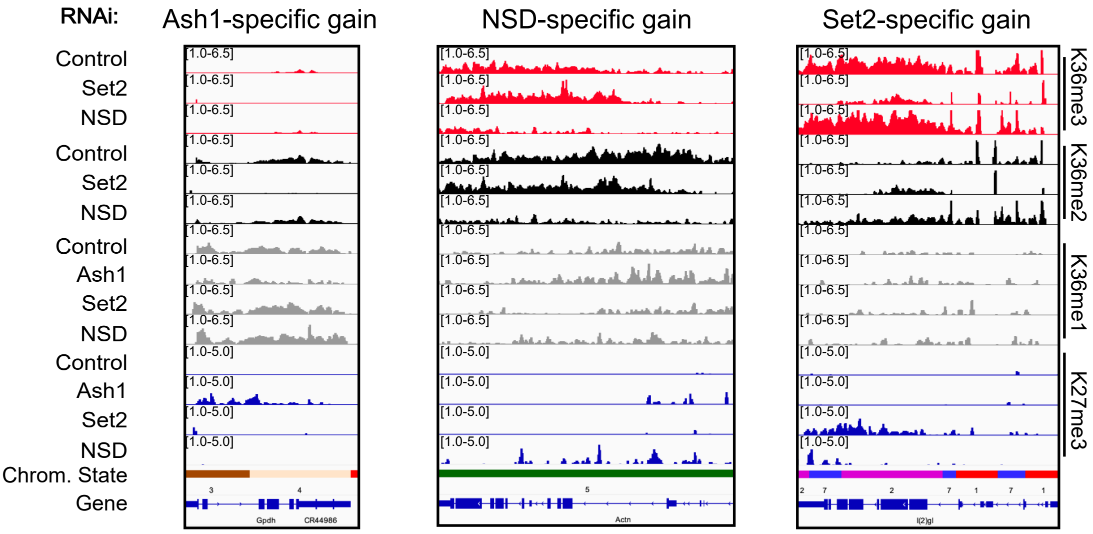

D

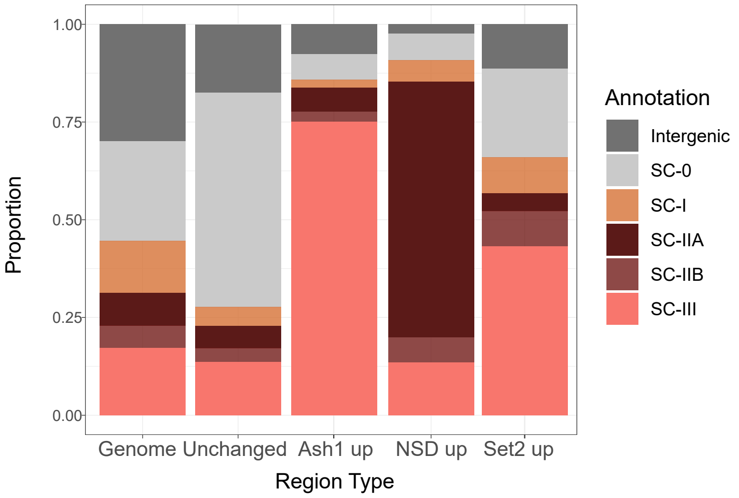

E

Bin Classification (change upon RNAi)	Major SC overlap	Number of genes (by bin overlap)	Number of genes (by top genic signal gain)
Ash1 gained	SC-III	111	79
NSD gained	SC-IIA	262	249
Set2 gained	SC-III	16	14

F

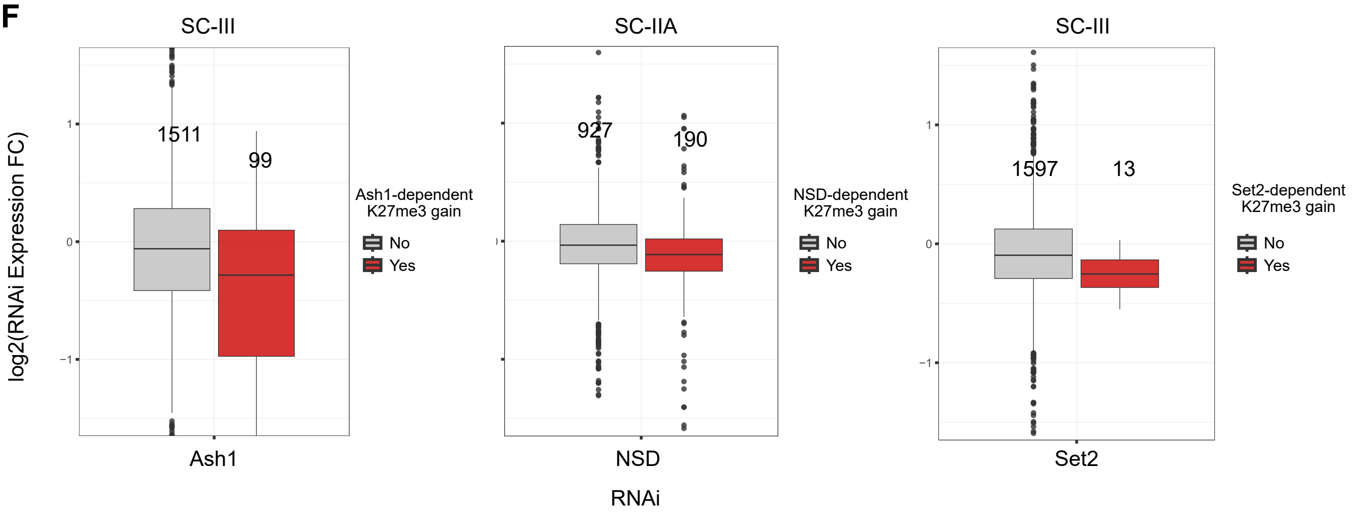

Supplementary Figure S9 : Reduction of K36me upon K36 HMT RNAi directly leads to gain of K27me3 at a subset of genes

A) Table summarizing changes in heterochromatin distribution in 5-kb bins (Left: H3K27me3; right: H3K9me2) using z-score approach described in schematic Figure 3A. Bins were classified as 'Lost' or 'Gained' upon RNAi of indicated factor based on custom thresholds (see Methods). Bins that don't change in any RNAi condition were classified as 'Unchanged'. n= Number of bins. N (Number of Biological Replicates) = 1

B) Venn Diagrams representing overlap between K27me3 bins identified as 'Gained' in different RNAi conditions

C) Genome browser profiles for representative regions that form *de novo* K27me3 domains upon RNAi of indicated factor. The histone modification that was analyzed by chromatin immunoprecipitation is indicated on right side.

D) Stacked barplots representing classification of bins where K27me3 was gained ('up') upon HMT RNAi as 'Intergenic' or belonging to Superclusters 0-III based on overlap of genomic coordinates. The 'Genome' bar indicates the proportion of genome covered by each annotation. The distribution of 'Unchanged' bins serve as reference. The total number of bins corresponding to the each bin type is provided in table from Supplementary Figure S9A.

E) Table representing the major gene supercluster overlapped by the 'Gained' K27me3 bins upon different RNAi (as inferred from above barplot) as well as the number of genes falling into each category defined by two different approaches (see Methods).

F) Boxplots representing gene expression changes for genes belonging to corresponding superclusters (as inferred from panels D,E) that gain K27me3 (red box) upon RNAi of indicated HMT. Expression changes for genes that belong to the same supercluster but do not gain K27me3 (gray box) serve as reference. Numbers displayed above the boxes correspond to number of genes with valid RNA-seq log2 Fold Change values. Note that the discrepancy in the number of genes displayed over red boxes compared to numbers represented in table (by bin overlap) in Supplementary Figure 9E arises due to filtering of silent genes (zero RNA-seq reads in all biological samples) for differential gene expression analyses.

A predominant view regarding K36 methylation function is that it serves to counteract the facultative heterochromatin mark K27me3. Previous studies in *Drosophila* have shown that Ash1 is the only methyltransferase whose loss of function leads to the classical homeotic transformation phenotypes (indicative of excessive *HOX* gene repression by Polycomb complex) (Dorafshan et al., 2019). However, more recent studies have shown that both Set2 and NSD depletion leads to gain of K27me3 in certain genomic regions (Depierre 2023). However, in these studies the changes in K27me3 weren't directly correlated to changes in K36 methylation, which we attempted to address using a limited dataset.

To this end, we generated K27me3 MNase-ChIP profiles in HMT RNAi S2 cells. Given the previous studies linking NSD and constitutive heterochromatin mark K9me2 (Saha et al. 2020, Alekseyenko et al., 2014), we also generated K9me2 profiles in NSD RNAi background. Since we had only one replicate for all conditions, we couldn’t use our previous approach using csaw to identify statistically different ChIP regions. Instead, we adopted the z-score based strategy shown in schematic Fig 3A and filtered stringently for bins showing the largest increases and decreases (see Methods for details) (Supplementary Figure S9A).This analysis suggests that while the majority of the K27me3 marked domains remain unchanged, a considerable fraction of bins show gains in K27me3 in all three HMT RNAi backgrounds. On the other hand, loss of NSD had negligible effects on K9me2 distribution. Given that the decreases in K27me3 are likely indirect, we instead focussed on characterization of bins that show strong increases. Of note, these newly formed domains still have considerably lower K27me3 signal compared to 'Unchanged' domains (data not shown), suggesting incomplete K27me3 deposition which may be due to residual K36me left in our RNAi approach. An overlap analysis of the increasing bins across the three different RNAi conditions showed minimal overlap, suggesting that all three methyltransferases counteract Polycomb deposition in distinct genomic regions (Supplementary Figure S9B). Genome browser profiles for representative regions are shown in Supplementary Figure S9C. Interestingly, note that the region that gains K27me3 in an Ash1 RNAi-specific manner (left) also contains Set2-dependent K36me2/3. Despite strong reduction in K36me2/3 upon Set2 RNAi in this region, there is no gain of K27me3 suggesting that Ash1-dependent K36me1 is critical to counteract K27me3 in this specific context.

We then assigned these bins as intergenic or genic SC 0-III depending on their genomic overlap and visualized the distribution as barplots. This exercise confirms that the majority of the bins gaining K27me3 upon Ash1 and NSD RNAi are located within genes of their respective superclusters i.e. SC-III and IIA respectively. Of note, we observe no encroachment of K27me3 into NSD-dependent heterochromatic genes (SC-IIB) or intergenic regions upon NSD RNAi, suggesting that NSD-dependent K36me2/3 is dispensable for segregation of facultative and constitutive heterochromatin. Interestingly, bins that gain K27me3 in a Set2-dependent fashion are also enriched on SC-III (but not SC-I) genes, likely unique genes that contain Set2-dependent K36me2 within SC-III which don’t gain K27me3 upon Ash1 RNAi. The number of genes spanned by these bins are summarized in Supplementary Figure S9E. We emphasize that only a fraction of supercluster genes gain K27me3, suggesting that Polycomb repression is not the default state upon loss of K36me. It’s also unclear why SC-I genes do not show a gain of K27me3; this may be due to presence of the ‘intermediate’ K36me2 which can also inhibit enzymatic activity of E(z) and/or lack of necessary factors to target Polycomb elements to housekeeping genes. We favor the second hypothesis as even SC-I genes which show near complete turnover of K36me2/3 upon Set2 RNAi do not gain K27me3 (data not shown).

Another readout for gain of genic K27me3 is the reduction of gene transcripts due to the repressive nature of the mark. To explore this, we used the previously mentioned RNA-seq datasets and compared the transcriptional change of genes that gain K27me3 in a particular RNAi condition compared to the rest of the genes within their respective superclusters. This shows that genes that gain K27me3 show a slight propensity to be downregulated compared to genes that do not gain K27me3. Taken together, these observations argue that all three methyltransferases independently counteract K27me3 deposition predominantly at a subset of developmental genes. While these observations are generally explainable by a mechanism involving direct inhibition of PRC2 by H3K36 methylation, our data do not preclude the possibility that these HMTs may counteract PRC2 by locally methylating a substrate other than H3K36 which may be addressed by future studies.

Supplementary Figure S10

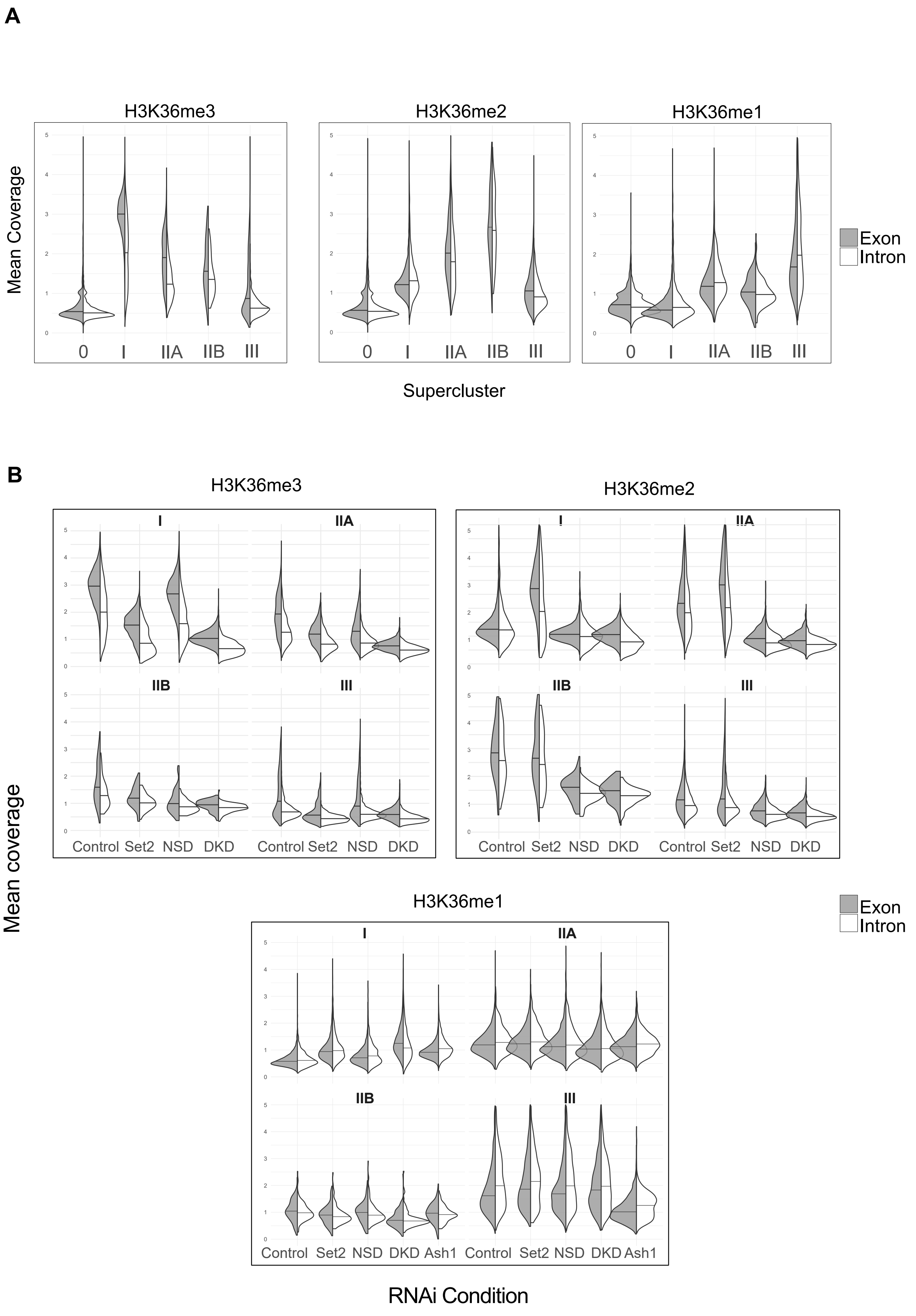

Supplementary Figure S10

Supplementary Figure S10: K36 methyltransferases show distinct preferences towards exons and introns.

A) Split violin plots representing distribution of H3K36me3/2/1 ChIP signal in introns (white) and exons (gray) of genes in Control RNAi within each supercluster.

B) Split violin plots representing changes in distribution of H3K36me3/2/1 ChIP signal in introns (white) and exons (gray) of genes in Control and HMT RNAi within each supercluster.

Supplementary Figure S11

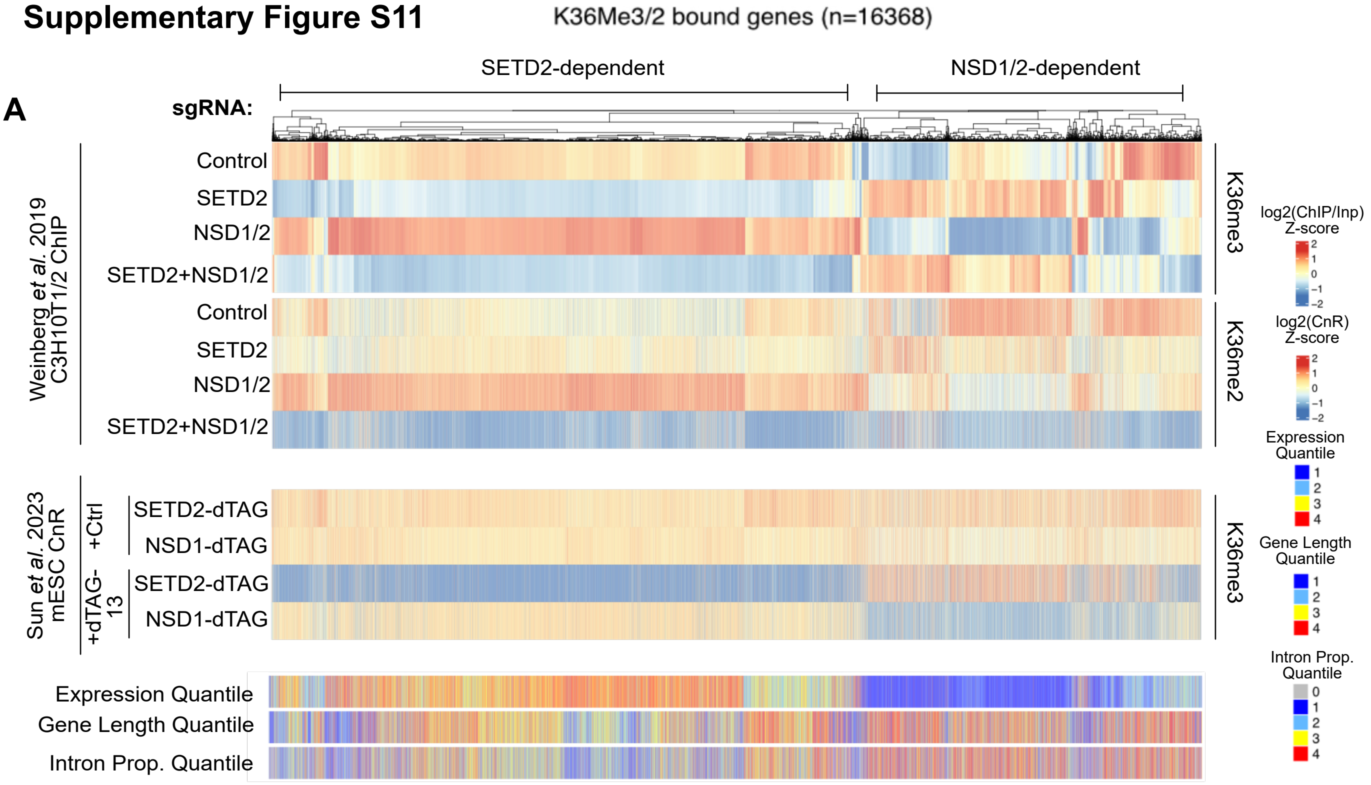

Supplementary Figure S11: Set2/NSD-dependent methylation patterns are conserved in mouse cells.

A) Top: Reanalysis of primary data from Weinberg et al., 2019. Clustered heatmap representing gene body averaged ChIP signal for H3K36me3/2 (indicated on right) in Control or HMT Knockout background (indicated on left) mouse C3H10T1/2 cells. Only genes overlapping at least one of K36me2/3 peaks in Control cell line (n=16368) were used for clustering. Values were standardized for each gene (by subtracting mean and dividing by standard deviation) across KO conditions separately for K36me2/3 before clustering.

Middle: Reanalysis of primary data from Sun et al., 2023. Heatmap representing gene body averaged CUT&RUN signal for H3K36me3 in untreated or dTAG-13 treated HMT degron tagged mESC cells. Values were standardized across treatment conditions and are ordered based on the hierarchically clustered ChIP heatmap.

Bottom: Heatmaps representing Quantiles of Expression, Gene Length and Intron Proportion ordered based on the ChIP heatmap (Quantile value 4=highest, 1=lowest; additional quantile category of 0 for Intron Prop. is to label genes with no introns). Expression data was obtained from Wang et al., 2019

The existence of NSD-dependent K36me3 domains was recently observed in mice (Barral et al., 2022). To verify if the genic methylation patterns that we observe are also conserved in mammals, we reanalyzed 2 additional recently published datasets (Weinberg et al., 2019; Sun et al., 2023). We first performed clustering analysis of genes under K36me2/3 peaks using H3K36me2/3 ChIP data from mouse C3H10T HMT Knockout (KO) cells (Weinberg et al., 2019) and correlated the clusters to H3K36me3 Cut&Run signal from mESC HMT-dTAG degron cells (Sun et al., 2023) as well as Gene Expression, Gene Length and Intronic Proportion Quantiles (Figure 5D). Of note, in this specific heatmap H3K36me2/3 signal was standardized (by subtracting the mean and dividing by standard deviation) across HMT perturbation conditions to robustly cluster genes which show similar patterns and thus doesn’t reflect absolute ChIP/CnR signal values. But overall, the absolute K36me3 signal at NSD1/2-dependent genes is about 3-10 fold lesser than SETD2-dependent genes depending on the mapping methodology used (as CnR strongly underestimates H3K36me3 signal at NSD-dependent genes).

We clearly observe two distinct responses in the HMT KO/dTAG-13 treated cells where SetD2 deposits H3K36me3 independent of NSD1/2 at highly expressed, short, exonic genes while NSD1/2 catalyzes H3K36me2/3 at poorly expressed, long, intronic genes. Intriguingly, the “see-saw” effect described in *Drosophila* is also apparent in these datasets where NSD1/2 is more active upon SetD2 depletion. Of note, the TKO me2/3 patterns are not a simple addition of the individual knockouts, which may hint at the involvement of other mammalian H3K36 HMTs (like SETD5 and/or NSD3) (Sessa et al, 2023) or altered demethylases localization. Importantly, these patterns are clearly visible across two very different perturbation strategies (Long-term Knockout vs Acute Inducible Degradation) and mapping methodology (ChIP vs CUT&RUN), supporting robustness of the phenomenon.

Supplementary Figure S12

A

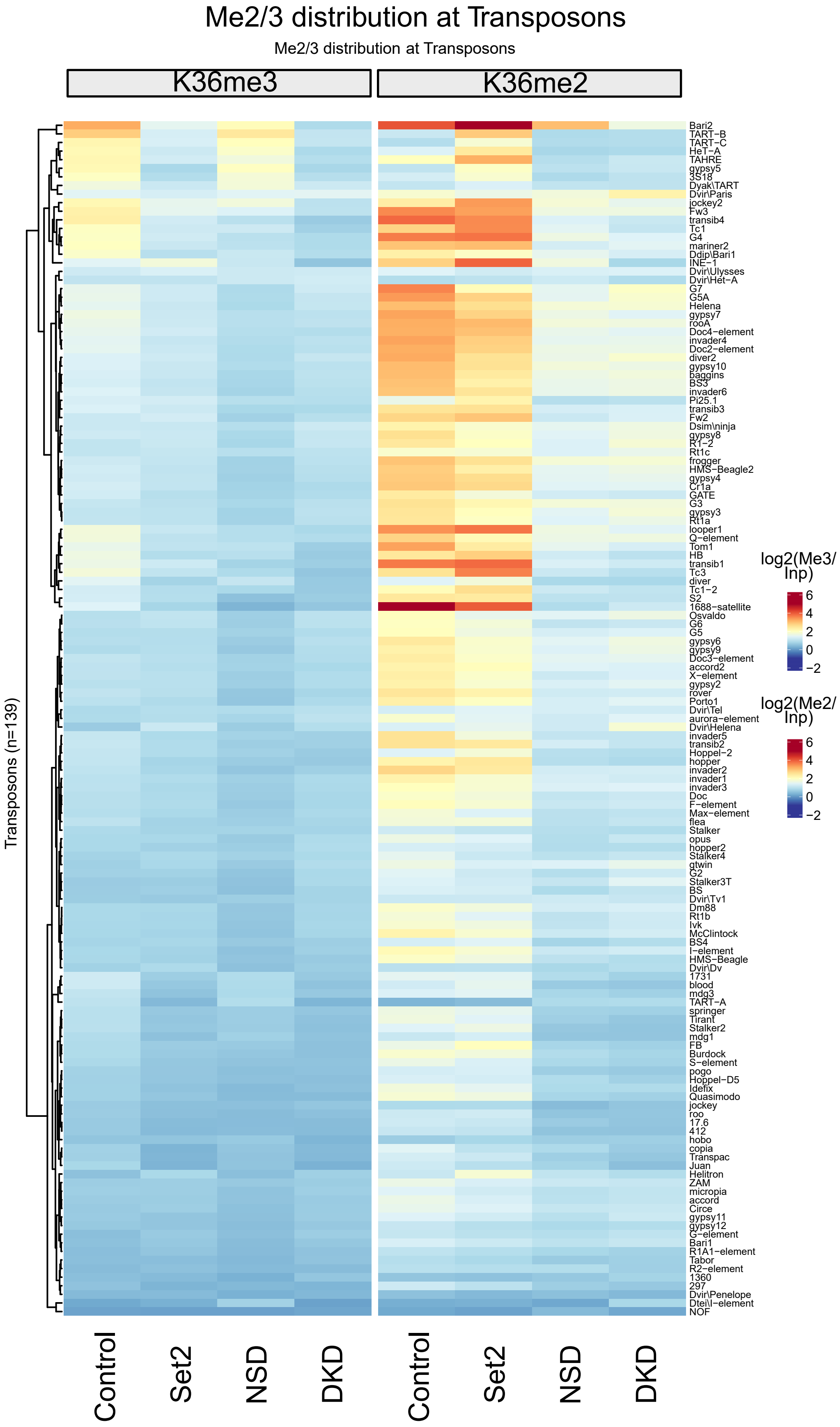

Supplementary Figure S12 : Set2 and NSD deposit K36me2/3 on distinct transposons.

A) Clustered Heatmap representing average K36me2/3 ChIP enrichment at 139 transposon families (names indicated on the right) in GST (Control), Set2, NSD or Set2+NSD (DKD) RNAi.

Supplementary Figure S13

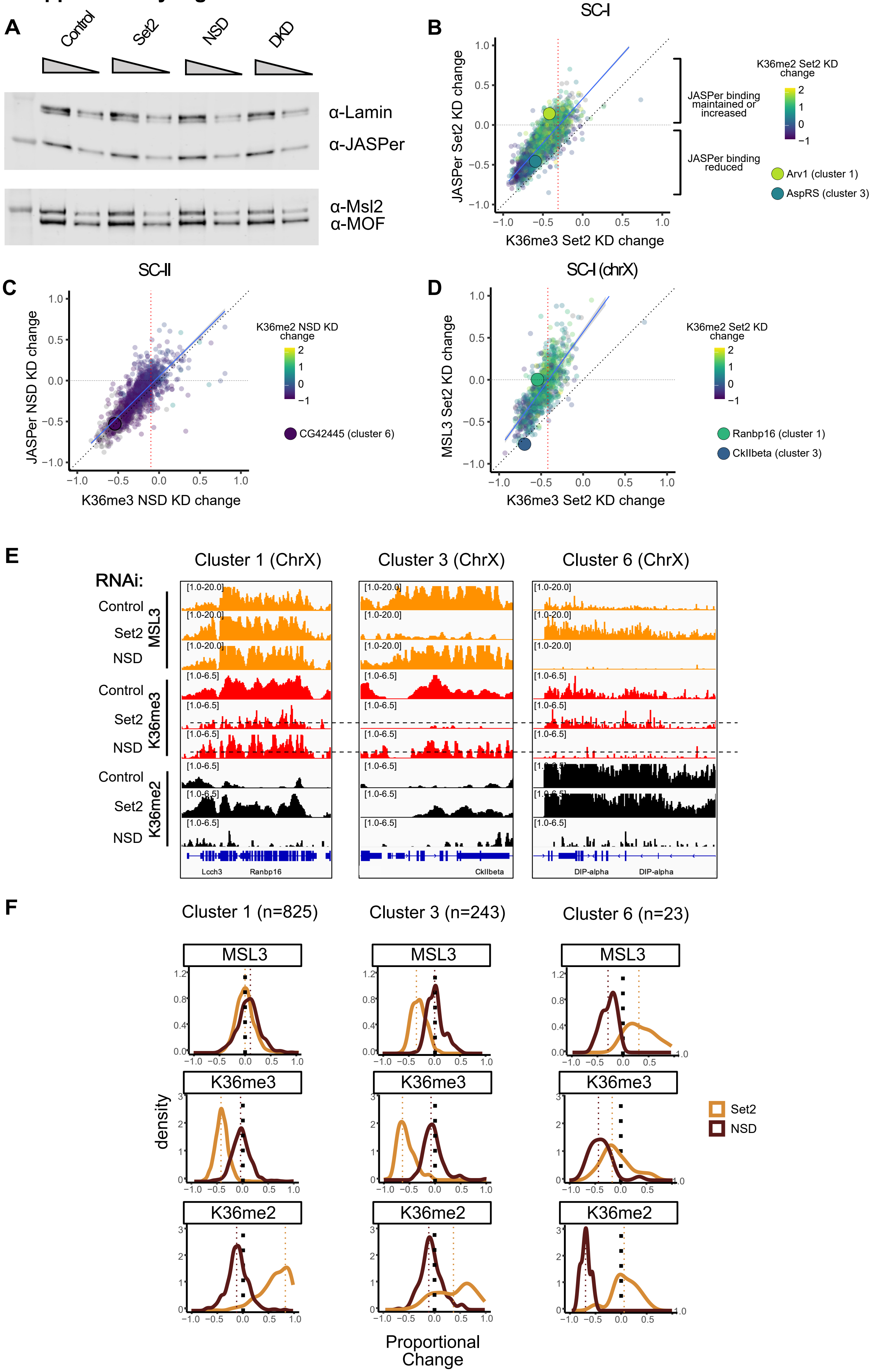

Supplementary Figure S13

Supplementary Figure S13: H3K36me reader binding is determined by threshold density of K36me2/3.

A) Western blot analysis using α-JASPer, α-Lamin, α-Msl2 and α-MOF antibodies and S2 whole cell extracts treated with Control, Set2, NSD or Set2+NSD (Comb) RNAi. Two dilutions (1x and 0.5x were loaded per sample)

B) Scatter plot representing JASPer Set2 RNAi proportional change (y-axis) as a function of K36me3 Set2 RNAi proportional change (x-axis) for SC-I genes. Each point represents a gene. The color of the points represents the K36me2 Set2 RNAi proportional change. The solid blue line denotes the population trendline. The red dashed line denotes the offset value of the trendline from the y=x line (black dashed diagonal line) and represents the magnitude of compensation by K36me2. Representative genes for clusters 1 and 3 as shown in Figure 6B are highlighted.

C) Scatter plot representing JASPer Set2 RNAi proportional change (y-axis) as a function of K36me3 NSD RNAi proportional change (x-axis) for SC-II genes. Each point represents a gene. The color of the points represents the K36me2 NSD RNAi proportional change. The solid blue line denotes the population trendline. The red dashed line denotes the offset value of the trendline from the y=x line (black dashed diagonal line) and represents the magnitude of compensation by K36me2. Representative genes for cluster 6 as shown in Figure 6B is highlighted.

D) Scatter plot representing MSL3 Set2 RNAi proportional change (y-axis) as a function of K36me3 Set2 RNAi proportional change (x-axis) for SC-I chrX genes. Each point represents a gene. The color of the points represents the K36me2 Set2 RNAi proportional change. The solid blue line denotes the population trendline. The red dashed line denotes the offset value of the trendline from the y=x line (black dashed diagonal line) and represents the magnitude of compensation by K36me2. Representative genes for clusters 1 and 3 as shown in Supplementary Figure 6F are highlighted.

E) Genome browser profiles for three distinct representative genes on the chromosome X highlighting differential response of MSL3 to Set2 RNAi and NSD RNAi within indicated clusters. RNAi condition and immunoprecipitation target indicated on left-hand side. The dotted line in K36me3 ChIP in Set2/NSD RNAi represents the threshold derived from scatter plot in Supplementary Figure S10B.

F) Proportional change density plots representing direction and magnitude of change in gene body average ChIP signal for K36me2/3 along with reader MSL3 for indicated representative clusters restricted to chromosome X. Dashed line indicates the median value for each curve.

Supplementary Figure S14

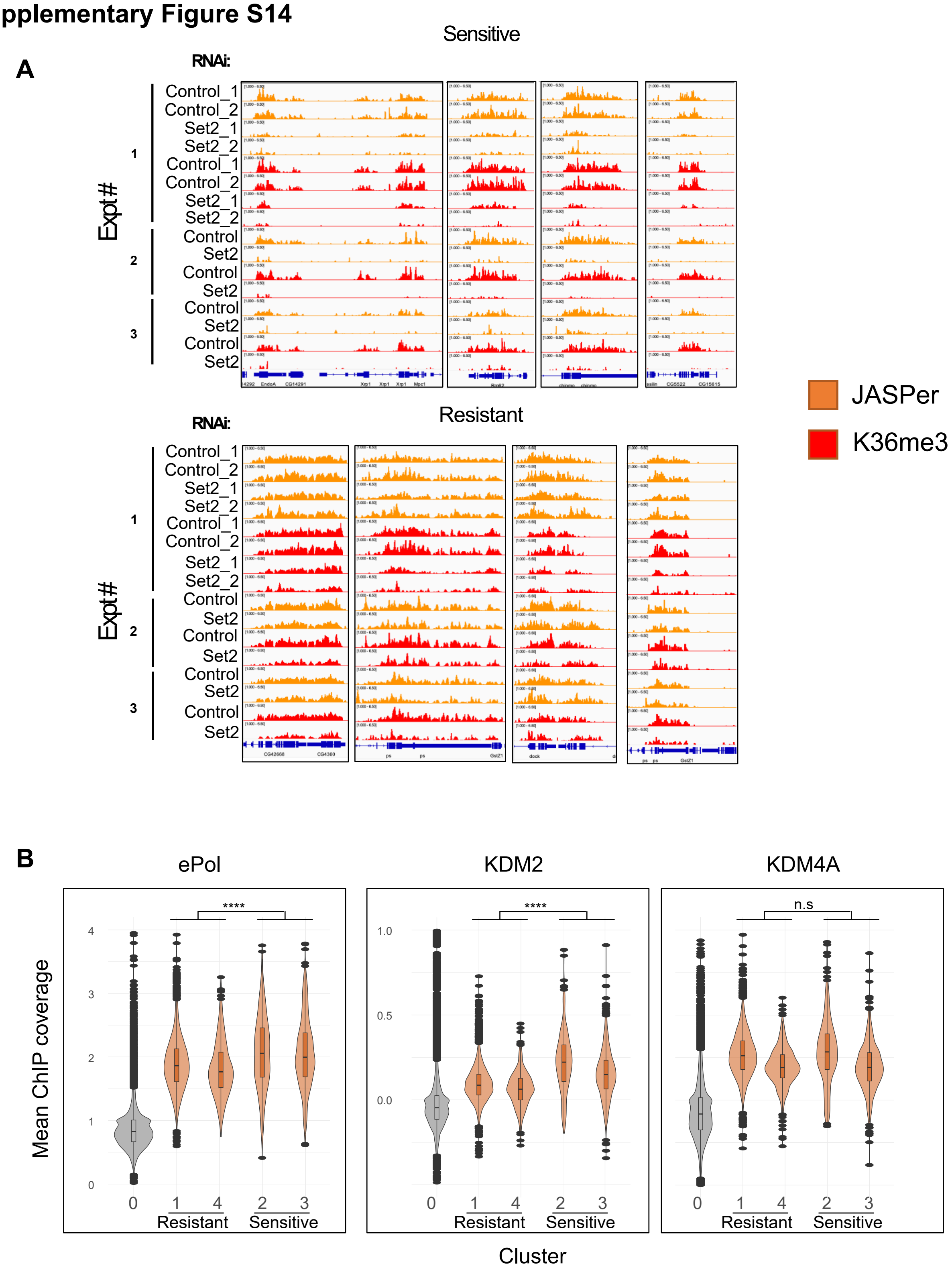

Supplementary Figure S14 : Factors involved in turnover of K36me2/3 may dictate 'resistant' or 'sensitive' outcomes upon Set2 RNAi.

A) Genome browser profiles for multiple genomic regions documenting reproducible 'sensitive' and 'resistant' phenotypes of K36me3/JASPer across replicate experiments of 7 day RNAi (Expt #1) or 10 day RNAi (Expt #2,3).

B) Violin plots representing gene-body average ChIP signal for HMT (ePol) and HDMs (KDM2 and KDM4A) for clusters within SC-I. Cluster 0 represents genes which lack any detectable H3K36 methylation and serves as a reference for zero signal/baseline. Definition of 'sensitive and 'resistant' clusters follows from main text. One-sided t-test was performed to identify factors selectively enriched in the 'Sensitive' clusters relative to the 'Resistant' clusters (**** denotes a significance value of $p < 0.001$, n.s=Not Significant).
